# Sustainable Protective Composite Textiles: Valorizing Hemp Hurd and Corn Stover Lignin via Electrospinning

**DOI:** 10.3390/polym18091124

**Published:** 2026-05-02

**Authors:** Dorota B. Szlek, Nara Han, Chang Geun Yoo, Margaret W. Frey

**Affiliations:** 1 Department of Human Centered Design, Cornell University, 37 Forest Home Drive, Ithaca, NY 14853, USA; margaret.frey@cornell.edu; 2 Department of Chemical Engineering, College of Environmental Science and Forestry, State University of New York, 1 Forestry Drive, Syracuse, NY 13210, USA; nhan@esf.edu (N.H.); cyoo05@esf.edu (C.G.Y.)

**Keywords:** corn stover, hemp, lignin, CELF, fibers, composite, textiles, electrospinning

## Abstract

Valorization of abundant agricultural residues, particularly lignin, provides the opportunity to divert waste streams while enabling materials to inherently exhibit durable functionalities, including UV-blocking, antioxidant properties and water repellency. This study reports the side-by-side valorization of hemp hurd (HL) and corn stover lignin (CL), extracted using the CELF process, into electrospun lignin/nylon 6 nanofiber membranes, establishing how lignin botanical origin, molecular weight (M_w_), and blend ratio govern multifunctional performance relevant to protective membranes in textiles. Lignin–nylon 6 hydrogen bonding was regulated by the OH content and accessibility, M_w_, and purity, and influenced the functional properties of the fibers. While stronger in low-M_w_ nanofibers, these interactions were weakest in low-M_w_ HL samples due to the lowest purity, despite the highest OH content. Fibers with low-M_w_ lignin yielded finer, brittle fibers with higher UV blocking, whereas high-M_w_ fractions showed higher antioxidant performance due to decreased interactions with nylon 6. Overall, lignin/nylon 6 nanofiber membranes delivered biobased UPF 50+ performance, 55–61% antioxidant activity at the optimal concentration, and exhibited tunable water repellency via fraction selection and the blend ratio. In combination with a nanofiber architecture, these membranes can impart durable inherent functionality onto textile substrates without affecting their existing properties, including water vapor permeability, without the use of chemical finishing, while utilizing renewable resources from agricultural residues.

## 1. Introduction

Crop residues are promising resources for various biorefinery applications. Among crop residues, corn stover and hemp hurd were selected for the study due to the large corn crop production in the U.S., and recent research interest in hemp. In particular, hemp has been highlighted for various applications because of legal restrictions on crop cultivation in the U.S. until recently [1,2,3,4,5,6]. So far, the utilization of hemp hurd and corn stover residues has been limited to low-value applications, so alternative higher-profit valorization pathways for these biomass resources can accelerate and expand their markets.

The use of lignin in fibers has been explored at length in recent years due to its renewability and beneficial inherent properties, such as hydrophobicity, thermal stability, UV blocking, and excellent antioxidant performance [7,8,9,10,11,12,13]. These inherent multifunctionalities of lignin, when incorporated into materials, can impart durable performance without the need for additional chemical treatments. This is especially beneficial for textile applications, where the majority of functional performance originates from chemical post-treatments [14].

However, the heterogeneity and branching of lignin structures have been known to affect processability, hindering their applications [15,16], including in fibers. Solvent fractionation of extracted lignin based on solubility can address this challenge by improving its molecular weight distribution and homogeneity, thereby potentially enhancing the processability and predictability of performance, which was shown in our previous study [15]. In addition to molecular weight, lignin fractions originating from hemp hurd and corn stover will differ in purity, as well as OH group, subunit and linkage contents. Increasing the homogeneity of the lignin raw material can ultimately support its spinnability in blends and help standardize attributes, both of which will be necessary for reproducibility and scaled operations. As an example, aside from variation in functional performance, high weight-average molecular weight (M_w_) and high polydispersity (*Ɖ*) in lignin were reported to lead to issues with spinnability, while low-M_w_ lignin was correlated with lower thermal stability, making it unsuitable for melt-spinning applications [13].

Amongst different types of fiber formation processes, electrospinning is an electrically driven method, convenient at the laboratory scale, that offers the flexibility of spinning from solution for a range of polymers, including biopolymers such as lignin. This method is noted for the versatility of simple parameter adjustments, such as voltage polarity and magnitude, ambient conditions, distance to the collector, collector type, and flow rate, in any setting for optimal results in micro- and nanofiber synthesis [17,18,19]. Difficult-to-spin polymers, including blends and branched, amorphous polymers, such as proteins and lignin, have been successfully processed using electrospinning, which relies on a voltage gap between the spinning solution and the collector rather than the mechanical extension of that solution.

However, the poor mechanical performance of homopolymer lignin fibers remains a limitation of electrospinning neat lignin. While blending lignin with synthetic polymers, which serve as carriers in fiber spinning, has been successful, achieving good lignin miscibility with synthetic polymers remains difficult [20]. Among many synthetic polymers, nylon 6 is easily electrospun into nanofiber mats [21,22]. By co-dissolving lignin and nylon 6 easily spinnable solutions can be processed into fibers combining the beneficial properties of both polymers including sufficient physical properties, hydrophobicity, anti-microbial and UV-resistant performance. Nylon–lignin fiber and non-fiber composites have been shown to have good compatibility through strong hydrogen bonding interactions between the hydroxyl groups in lignin and the amide groups in nylon 6 [23,24,25,26], which can be further enhanced with the use of 1,1,1,3,3,3-Hexafluoro-2-propanol (HFIP) as a solvent. HFIP has the potential to enhance the supramolecular structure of lignin and easily dissolve both nylon 6 and lignin at room temperature without the need for derivatization [27,28]. The use of easily electrospun nylon 6 as a carrier polymer can be advantageous for synthesizing high-lignin-concentration nano/microfiber mats with minimal support from the carrier polymer, while retaining the inherent multifunctionality of lignin in fiber form. To achieve fully biobased fibers, biobased nylons and even nylon precursors generated through lignin conversion [29] can be used as alternative carrier polymers to optimize the composition further.

Herein, lab-scale electrospun nanofiber mats were produced with nylon 6 and fractionated lignins from hemp hurd and corn stover residues. The characteristics of lignin from both agricultural residues were investigated to understand their correlations with the performance and properties of lignin–nylon 6 fibers. The spinnability and functionality of the lignin/nylon fibers were also discussed.

## 2. Materials and Methods

### 2.1. Materials

Corn stover was provided by the University of California Riverside. Milled hemp hurd was purchased from Eaton Pet and Pasture. HPLC-grade MeOH was purchased from Fisher Chemical (Waltham, MA, USA); 1,1,1,3,3,3-hexafluoro-2-propanol (HFIP) and anhydrous 99.8% tetrahydrofuran (THF) from Thermo Fisher Scientific (Ward Hill, MA, USA); DPPH from Cayman Chemical Company (Ann Arbor, MI, USA); N, N-dimethylformamide (DMF) and acetic anhydride from Sigma-Aldrich (St. Louis, MO, USA); extra dry, stabilized 99.9% dichloromethane (DCM) from Acros Organics (Fair Lawn, NJ, USA; tetrahydrofuran (THF) from J.T. Baker (Phillipsburg, NJ, USA); ethanol from Deacon Laboratories Inc. (King of Prussia, PA, USA); pyridine from Honeywell (Houston, TX, USA); dimethyl sulfoxide (DMSO-d_6_) and chloroform-d (CDCl_3_-d), from Cambridge Isotope Laboratories, Inc. (Tewksbury, MA, USA); anhydrous pyridine, chromium(III) acetylacetonate (Cr(acac)_3_), endo-N-hydroxy-5-norbornene-2,3-dicarboximide (NHND), and 2-chloro-4,4,5,5-tetramethyl-1,3,2-dioxaphopholane (TMDP) from Merck KGaA (Darmstadt, Germany). Plain weave cotton fabric (110 gsm) and plain weave nylon 6.6 fabric (190 gsm) were purchased from Testfabrics, Inc. (West Pittston, PA, USA).

### 2.2. Extraction of Hemp Hurd and Corn Stover Lignins

For corn stover extraction, the co-solvent-enhanced lignocellulosic fractionation (CELF) process was conducted in a Parr reactor (Parr Instrument Co., Moline, IL, USA) at 160 °C for 30 min. A tetrahydrofuran (THF)-water mixture (2:1 weight ratio) and 1% sulfuric acid (dry biomass basis) were used as the solvent. The hemp hurd lignin was extracted using the CELF process in a Parr reactor at 170 °C for 30 min. For the solvent system, a THF–water mixture (1:1 weight ratio) and 0.36% sulfuric acid were used. Both hemp hurd and corn stover lignin were recovered from the processing liquor by filtration and washed with deionized water (DIW), then vacuum-dried at 40 °C.

### 2.3. Solvent Fractionation of Lignins

The corn stover lignin soluble–low (CLL) and insoluble–high (CLH) M_w_ fractions were obtained by stirring in 200-proof ethanol for 6 h. CELF-extracted hemp hurd lignin was fractionated with an ethanol–water mixture (7:3 volume ratio) with stirring for 6 h. The low and high M_w_ fractions were separated by centrifugation at 10,000 rpm for 20 min. The low-M_w_ fraction of hemp hurd lignin (HLL) was recovered by rotary evaporation, and the high-M_w_ fraction of hemp hurd lignin (HLH) was air-dried and further vacuum-dried at 40 °C.

### 2.4. Spinning Solution Preparation

Hemp hurd and corn stover soluble- and insoluble-lignin fractions were co-dissolved with nylon 6 in 1,1,1,3,3,3-hexafluoro-2-propanol solvent in the following weight ratios: 100:0, 95:5, 67:33, 33:67, and 0:100 lignin:nylon 6, at a total concentration of 10 wt.%. The CLL-to-nylon 6 at 95:5 and 67:33 ratios were also prepared at a 15 wt.% concentration to compare the spinnability and fiber diameter. Solutions were vortexed for 30 s and then placed on a shaker for 24 h before electrospinning.

### 2.5. Electrospinning Process for Lignin/Nylon 6 Nanofibers

Spinning parameters were optimized at a 2 mL/h flow rate, a 15 cm distance to the collector, and −20 kV voltage applied to the needle tip, with a grounded collector. The horizontal electrospinning setup included a Gamma High Voltage Research power supply and a Harvard Apparatus PHD/ULTRA syringe pump. BD syringes (5 mL) with a BD PrecisionGlide 21 G needle were used for solution loading and extrusion. The collector was a copper plate covered with aluminum foil and placed across from the syringe in a Plexiglas box. The electrospinning current was not independently measured and was governed by the applied voltage and solution conductivity. Relative humidity and temperature were recorded and ranged from 64.2 to 69.9% and 19.6–21.2 °C. Nanofiber mats were collected in triplicate from neat nylon 6 and nylon 6 blended with different lignin concentrations, as outlined in Table 1. The duration of each electrospinning run was 10 min.

### 2.6. Chemical Composition Analysis of Lignins

The chemical composition analysis of lignin was quantified according to the National Renewable Energy Laboratory protocol (NREL/TP–510–42618). Cellulose and hemicellulose were measured by high-performance liquid chromatography (HPLC, Agilent Technologies 1260 Infinity, Santa Clara, CA, USA). Acid-soluble lignin was determined by a Genesys 10S UV-Vis spectrophotometer, and acid-insoluble lignin was measured gravimetrically. All measurements were duplicated.

### 2.7. Nuclear Magnetic Resonance (NMR) Analyses of Lignins

Two-dimensional heteronuclear single quantum coherence (2D HSQC) NMR analysis was carried out on a Bruker AVANCE III HD 800 MHz NMR equipped with a TCI cryoprobe using deuterated dimethyl sulfoxide (DMSO-*d*_6_) as a solvent. The lignin samples were measured with 32 scans and a relaxation delay of 1.0 s. The spectral widths for ^1^H and ^13^C dimensions were 12 ppm and 220 ppm, respectively. Phosphorus NMR was conducted on a Bruker AVANCE III HD 600 MHz NMR with a 5 mm BBFO liquid nitrogen cold probe in triplicate. For sample preparation, chromium (III) acetylacetonate and endo-N-hydroxy-5-norbornene-2,3-dicarboximide (NHND) were dissolved in an anhydrous pyridine-deuterated chloroform mixture (1.6:1, *v*/*v*) to prepare an NMR solution. The vacuum-dried lignin sample was subsequently dissolved in the prepared solution. After adding 2-chloro-4,4,5,5-tetramethyl-1,3,2-dioxaphopholane (TMPD) as phosphorylation reagent, NMR measurement was conducted within 2 h using 64 scans and a 25.0 s relaxation delay. To quantify the hydroxyl group content, NHND was used as an internal standard.

### 2.8. Molecular Weight Analysis of Lignin

The M_w_, M_n_, and dispersity (*Ɖ*) of each lignin fraction were measured by gel permeation chromatography (GPC), following a procedure reported by Ryu et al. [30]. For the sample preparation, the lignin was acetylated with a pyridine–acetic anhydride mixture (1:1, *v*/*v*) for 24 h. The acetylated lignin was recovered by rotary evaporation, dissolved in THF, and filtered through a 0.45-μm PTFE syringe filter. An Agilent GPC SECurity 1200 system (Santa Clara, CA, USA) with three Styragel columns (HR 1, HR 3, and HR 4E) was utilized for GPC analysis with a Waters 2489 UV/Vis detector (Milford, MA, USA). THF was used as the mobile phase with a flow rate of 1 mL/min. Seven polystyrene standards (500–50,000 g/mol) were employed to construct the calibration curve.

### 2.9. Attenuated Total Reflectance (ATR)—Fourier-Transform Infrared (FTIR) Analysis

FTIR spectra of CLL, CLH, HLL, HLH, and their fibers, as well as N6 fibers, were collected in triplicate using a spectrometer FTIR-ATR Perkin Elmer Spectrum (PerkinElmer, Waltham, MA, USA). Triplicate measurements were collected in the wavelength range from 4000 to 600 cm^−1^ at a data interval of 0.5 cm^−1^, with a resolution of 4 cm^−1^ over a total of 16 scans. Spectra were baseline-corrected in OriginPro software (2023b 64-bit SR1), then normalized with the integrated C–H stretching vibrations peak (3000–2835 cm^−1^) area. Deconvolution of the OH region was performed in OriginPro using second-derivative curve-fitting with a Gaussian peak type, fixed (y_0_) = 0, and a minimum peak height of 3%. Outputs included the peak center wavenumber and the integrated peak area (Absorbance·cm^−1^).

### 2.10. Thermogravimetric Analysis (TGA)

TGA was performed on the CLL, CLH, HLL, and HLH powders, as well as on nylon 6 and lignin/nylon 6 fibers using a TGA Q500, V20.10 Build 36 from TA instruments (New Castle, DE, USA). The samples were placed in a platinum pan, equilibrated to 30 °C, and then heated at a rate of 20 °C/min with flow rates of 40 mL/min balance nitrogen gas and 60 mL/min sample nitrogen gas. All fibers were heated to 800 °C, and the starting weights of the samples were between 0.87 mg and 1.36 mg for the fibers and 4.99 mg and 6.23 mg for the lignin raw materials. The onset degradation temperatures, total weight loss, and residual char of the fibers were studied and compared between the different samples.

### 2.11. Mechanical Strength Measurement of Fibers

A TA Instruments Dynamic Mechanical Analysis (DMA) Model Q800DMA (New Castle, DE, USA). was used to obtain the stress/strain curve of the CLL, CLH, HLL, HLH, and nylon 6 fiber samples that were successfully mounted in the clamp. Nanofiber mat strips with an average length of 7.89 mm, average width of 6.45 mm, and average thickness of 0.09 mm were cut, and the thickness of each tested strip was measured at three points and then averaged. From those data, the ultimate tensile strength, Young’s modulus, elongation at break, stiffness, relaxation modulus, displacement, and creep compliance were obtained. Testing was performed in triplicate at a ramp force of 0.5 N/min to 18.0 N.

### 2.12. Scanning Electron Microscope (SEM) and Fiber Diameter

Micrographs of CLL, CLH, HLL, HLH, and nylon 6 fibers were obtained with a Zeiss Gemini 500 SEM (Oberkochen, Germany) with InLens and HE-SE2 detectors at an acceleration voltage of 3 kV and a working distance of 4 mm. Prior to SEM analysis, each sample was coated with Au/Pd for 90 s using a Quorum SC7620 sputter coater instrument (Laughton, East Sussex, UK) under vacuum. The fiber diameter was measured during imaging using the Zeiss Gemini 500 measurement tool based on 10 different fibers within the same electrospun mat sample. The average fiber diameter and standard deviation were calculated for each sample.

### 2.13. Antioxidant Activity of Lignin/Nylon 6 Fibers

The antioxidant activities of CLL, CLH, HLL, and HLH fiber samples were evaluated by measuring the reduction rate of the 2,2-diphenyl-1-picrylhydrazyl (DPPH) radical in the presence of the antioxidants by measuring UV-Vis absorbance using a BioTek Synergy Neo2 multimode microplate reader (Santa Clara, CA, USA). Additionally, the antioxidant activity of ascorbic acid was measured as a positive control and that of N6 fibers as a negative control. A DPPH solution in methanol (MeOH) was prepared right before beginning the assay, and 2 mL of a 60 µM DPPH solution in MeOH was loaded into each vial containing 2 mg of fibers. Samples were incubated at room temperature under light protection. Afterwards, 100 µL of each solution was pipetted into 3 wells in 96-well microtiter plates with lids. Absorbance measurements were taken in triplicate at 517 nm at 2 timepoints: immediately after (within 10 min) addition of the DPPH solution to the fibers and 24 h after assay initiation. The antioxidant activity of the samples was calculated using Equation (1), based on the residual DPPH content.

(1) $$\mathrm{Antioxidant}\;\mathrm{Activity}\;(\%)=\frac{A_{0}-A_{1}}{A_{0}}\times 100$$

where

A_0_ is the absorbance of the neat DPPH solution (control)

A_1_ is the absorbance of the DPPH/fiber solution after assay initiation

### 2.14. Water Vapor Permeability of Lignin/Nylon 6 Fibers

The water vapor permeability (gsm/day) and the water vapor permeability index were evaluated following the BS 7209.1990 [31] specification for water vapor permeable apparel fabrics. First, different lignin/nylon 6 solutions and a neat nylon 6 solution were electrospun in triplicate for 30 min each onto plain-weave 100% cotton fabric and onto plain-weave 100% nylon 6.6 fabric 83 mm diameter circular substrates, following the electrospinning method from Section 2.5. The mass and thickness of the neat fabric samples were measured before and after electrospinning. Uncoated substrates of cotton and nylon fabric, as well as aluminum foil, were measured as controls. Then, electrospun fabric samples were conditioned for 2 h in a laboratory with a controlled temperature atmosphere. The samples were then mounted on corrosion-resistant open dishes with wire supports and cover rings with the help of adhesive cement and adhesive tape to prevent uncontrolled vapor transmission outside of the sample surface area. Afterwards, the samples mounted on the open dishes were placed on a rotating SDL Atlas M261 water vapor permeability tester turntable (Rock Hill, SC, USA) for 24 h. The mass of the ensemble was measured before and after the 24 h rotation. The water vapor permeability of the neat substrate samples and nanofiber/substrate samples was calculated using Equation (2):

(2) $$\mathrm{Water}\;\mathrm{Vapor}\;\mathrm{Permeability}\;(\mathrm{gsm}/\mathrm{day})\;=\;\frac{24\;\times \;M}{A\;\times \;T\;}$$

where:

T is the time between successive weightings of the assembly (in h).

M is the loss in mass of the assembly over the time period T (in g).

A is the area of the exposed test fabric (equal to the internal area of the test dish) (in m^2^).

The water vapor permeability index of the nanofiber/substrate samples was calculated using Equation (3):

(3) $$\mathrm{Water}\;\mathrm{Vapor}\;\mathrm{Permeability}\;\mathrm{Index}\;(\%)\;=\;\frac{{WVP}_{f}}{{WVP}_{r}}\times 100$$

where:

WVP_f_ is the mean water vapor permeability of the fabric under test.

WVP_r_ is the water vapor permeability of the reference fabric.

### 2.15. Water Contact Angle Analysis of Neat Nylon6 and Lignin/Nylon 6 Fibers and Fiber-Coated Cotton and Nylon Fabrics

Water contact angle measurement was carried out using a Ramé-Hart 500 contact angle goniometer/tensiometer with DROPimage Advanced (p/n 500-U4) (Ramé-Hart Instrument Co., Succasunna, NJ, USA). A 10 µL droplet of deionized water was deposited on the surface of each sample using an automated dispensing system fitted with an 18-gauge needle. Triplicate measurements were taken 10 s after deposition by applying the water droplet in three different areas of each sample. Contact angle analysis was performed using ImageJ (v1.54g), and the average contact angle was calculated for each sample.

### 2.16. UV Transmittance of Neat Nylon 6 and Lignin/Nylon 6 Fiber Membranes

The UV blocking properties of the fiber mats were investigated using a Cary 5000 UV/Vis/NIR spectrophotometer (Agilent Technologies, Santa Clara, CA, USA) via Cary WinUV software (v6.5). The diffuse transmittance (%) of mounted samples was measured in the wavelength range of 280–400 nm with a 4 mm aperture, a 312 nm source changeover, a 0.4 s averaging time and a 2 nm spectral bandwidth. The % transparency of the film in the ranges 280–315 nm (UV-B) and 315–400 nm (UV-A) was determined. Ultraviolet Protection Factor (UPF) values were calculated from the measured transmittance according to AATCC TM183-2020e [32].

### 2.17. Electrical Conductivity

Electrical conductivity of nylon 6 and lignin/nylon 6 solutions, as well as the neat solvent as a control, was measured using an Oakton Instruments PC2700 benchtop meter (Vernon Hills, IL, USA) with a conductivity probe.

### 2.18. Statistical Analysis

Statistical significance of all results was analyzed using ANOVA and paired two-sided *t*-tests at a 95% confidence level, comparing two arrays of mean values from the same sample set. In many cases, the indication of low significance stemmed from the small size of the population tested.

Multiple linear regression was fitted in OriginPro 2023b (64-bit) SR1. Neat lignin measurements (such as phenolic OH content) were normalized to the composite mass relative to the respective concentrations and scaled in correlations with the physicochemical properties of samples for accuracy.

## 3. Results and Discussion

### 3.1. Characteristics of the Solvent Fractionated Lignins

Before applying the fractionated lignins, their chemical composition (i.e., purity), physicochemical properties, such as molecular weight, interunit linkage content, and hydroxyl group content, were analyzed (Figures S1 and S2; Tables S1 and S2). Overall, the fractionated lignins showed 88–97% purity, with only a small amount of carbohydrates from cellulose and hemicellulose; therefore, these lignins were used without further purification (Table 2). The fractionated lignins (i.e., CLL, CLH, HLL, and HLH) had a relatively uniform molecular weight distribution (*Ɖ* = 1.7–2.5). The M_w_ of CLH and HLH was 4623 and 8040 g/mol, respectively, which was much higher than that of the soluble fractions (CLL with 1325 g/mol and HLL with 3401 g/mol), indicating that solvent fractionation successfully separated the hemp hurd and corn stover lignin by molecular weight.

**Figure 1:**
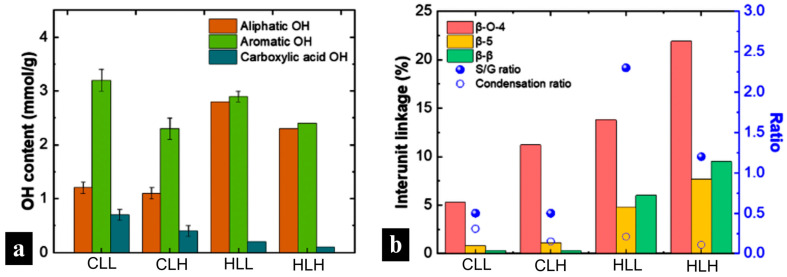
(**a**) Hydroxyl contents of lignin fractions. (**b**) Interunit linkage, S/G ratio, and condensation ratio of lignin fractions.

Hydroxyl groups in the lignin structure play an important role in its material applications because these functional groups are potential binding sites to the copolymer in the composite matrix. As Figure 1 shows, low-M_w_ lignin fractions (CLL and HLL) had higher total OH contents. In particular, low-M_w_ corn stover lignin had higher aromatic OH contents.

### 3.2. Lignin/Nylon 6 Spinning Process Optimization

#### 3.2.1. Spinnability of Lignin/Nylon 6 Solutions

Attempts at electrospinning corn stover lignin dissolved in solvents including tetrahydrofuran (THF) and DMF/dichloromethane (DCM), previously used in other studies [15,18,33], resulted in the successful formation of neat lignin microfiber mats (Figure S3). Nevertheless, the brittleness of the obtained mats made them unusable, leading to the decision to blend lignin with nylon 6, a synthetic polymer with excellent spinnability and mechanical properties, prior to electrospinning.

Electrospinning of all lignin/nylon 6 solutions resulted in the successful collection of nanofiber mats. HFIP is a good solvent for both lignin and nylon 6, eliminating the need for multiple co-solvents, thermal treatments, or derivatization. Because of the presence of a carrier polymer, nylon 6, the spinnability of lignin/nylon 6 solution blends was not significantly affected by the lignin source (corn stover or hemp stalks) or whether the low- or high-M_w_ lignin fraction was selected. However, the selection of the lignin fraction significantly affected the properties of the fiber mats. Insoluble fractions aggregated in solution (Figure S4a) consistent with higher M_w_ and a greater degree of branching [34], while soluble lignin fractions showed excellent solubility in the solvent (Figure S4b).

In terms of spinning conditions, neat nylon 6 showed the highest yield on a positive voltage setup, opposite to lignin, which showed a much higher yield (based on a comparison of the intensity of the brown lignin color) on a negative vs. positive voltage setup (Figure S5b). This is due to the presence of negatively charged functional groups, such as phenolic and carboxylic acids, in lignin, supporting the repulsion of the solution jet at the needle tip (Figure S5a). In lignin/nylon 6 nanofiber mats collected using the (+) voltage setup, the layout of the collected fibers follows that of neat nylon 6 using the same voltage, reconfirming the role of nylon 6 as a carrier polymer.

#### 3.2.2. Electrical Conductivity of Lignin/Nylon 6 Solutions

**Figure 2:**
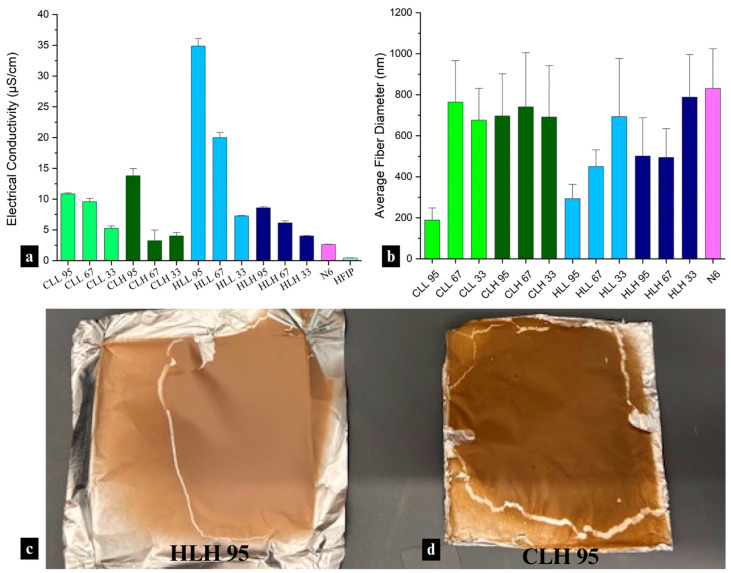
(**a**) Electrical conductivity of nylon 6 solution, all lignin/nylon 6 solutions, and neat HFIP. (**b**) Average fiber diameter of all lignin/nylon 6 and neat nylon 6 fibers; and images illustrating color variation in lignin/nylon 6 nanofiber mats using (**c**) hemp hurd lignin and (**d**) corn stover lignin.

Blended solutions containing soluble lignin fractions exhibited higher electrical conductivity than insoluble fractions (Figure 2a), which is consistent with the higher abundance of OH groups, thereby enhancing ionic transport (*r* = 0.54; *p* < 0.05). The electrical conductivity of these solutions also showed an inverse relationship with M_w_ (*r* = −0.24; *p* < 0.005) and *Ð* (*r* = −0.42; *p* < 0.005). These relationships were previously demonstrated in organosolv lignin [35].

In terms of biomass origin, low-M_w_ hemp hurd lignin showed superior electrical conductivity to low-M_w_ corn stover lignin in solution, which corresponds with a significantly lower condensation ratio, pointing to better solubility, and more exposed functional groups expected to increase ionic conductivity (*p* < 0.001) [36].

While electrical conductivities of the solutions did not govern spinnability, only those with the highest conductivity (HLL-containing mats) showed a correlation with fiber diameter (*r* = −0.99, respectively; *p* < 0.1), with higher conductivity, together with a higher lignin:nylon 6 composition ratio, driving smaller average fiber diameters in nanofiber mats. The electrical conductivity of mats containing other lignin fractions was too low to be significantly impactful in electrospinning.

### 3.3. Lignin/Nylon 6 and Neat Nylon 6 Fiber Characterization

#### 3.3.1. Visual Appearance, Morphology and Fiber Diameter

Hemp hurd lignin/nylon 6 and corn stover lignin/nylon 6 fiber mats differed visually, with hemp lignin samples showing a pink-toned brown hue (Figure 2c) and corn stover lignin samples—a yellow/orange-brown hue (Figure 2d). This difference in appearance correlates to the chromophoric group content in both lignins, driven by the higher condensation ratio, as well as a higher abundance of *p*-coumarate and ferulate groups responsible for the yellow hue in corn stover lignin (Table S2). Fiber mat color also influences its functionality, specifically UV-blocking performance.

**Figure 3:**
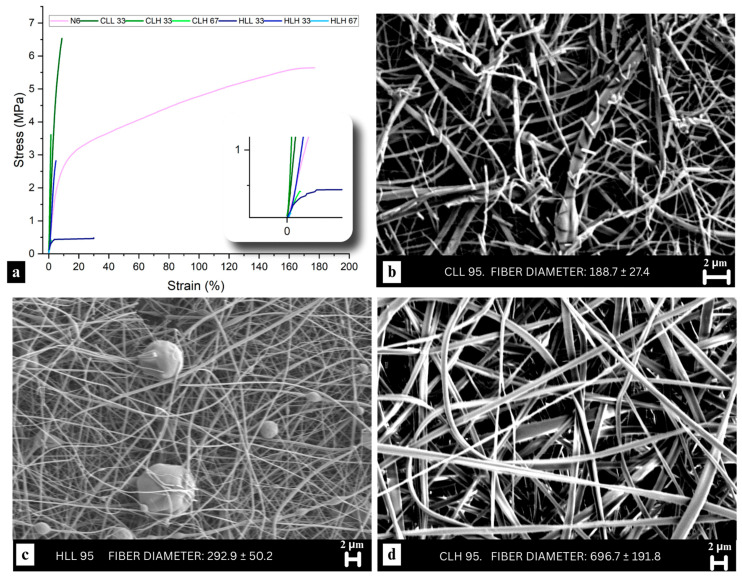
(**a**) Stress–strain curves of all tested lignin/nylon 6 and neat nylon 6 fibers. Curve colors correspond to the different samples, as indicated in the legend. SEM micrographs comparing morphology and brittleness in 95% (**b**) CLL, (**c**) HLL, and (**d**) CLH fibers.

Overall, all fibers had a smooth morphology, and corresponding micrographs can be found in Figure 3b–d and Figure S6. However, more breakage and apparent brittleness were observed for the higher lignin concentration fibers. This was especially evident in the CLL67 and CLL 95 samples (Figure 3b and Figure S7a,b). In comparison, while the HLL 67 and HLL 95 samples did not show as much breakage or brittleness due to the relatively higher lignin M_w_ (Figure 3c and Figure S6), some less severe breakage of the overall mat was observed (Figure S7c). On the other hand, CLH 95 fibers (Figure 3d), whose M_w_ was higher compared to HLL 95, showed no breakage. Furthermore, HLH 33 micrographs revealed possible phase separation between lignin and nylon 6, with a lignin core and a nylon 6 sheath (Figure S8a,b), a behavior previously observed with PLA [15].

Presence of lignin at different ratios with nylon 6 did not have a major impact on fiber diameter (*p* < 0.05) except for soluble lignin at the highest concentrations, which had a significantly smaller fiber diameter (Figure 2b). This is reflective of the lowest M_w_ of the soluble lignin fractions and points to the role of nylon 6 as the carrier polymer driving fiber formation and dictating the diameter based on its content in the blend.

Differences in fiber diameter also serve as initial indicators of interactions between lignin and nylon 6. Control solutions of neat nylon 6 in HFIP were electrospun at 0.5 wt.%, 3.3 wt.%, and 6.7 wt.% (actual nylon 6 concentrations in nylon 6/lignin 95, 67, and 33% samples) to assess the spinnability of nylon 6 without the addition of lignin. At 6.7%, the average fiber diameter of neat nylon 6 fibers was smaller than that of lignin/nylon 6 fibers at the same concentration (630 ± 155 nm). At 3.3%, the average fiber diameter was smaller than that of corn stover lignin/nylon 6 fibers but larger than that of hemp hurd lignin/nylon 6 fibers (540 ± 128 nm). At the lowest concentration of 0.5%, only spray was observed on the collector, whereas nanofiber mat formation was observed in lignin/nylon 6 fibers at the same concentration, confirming interactions between lignin and nylon 6.

#### 3.3.2. FTIR Analysis of Intra- and Intermolecular Interactions in Lignin/Nylon 6 Nanofibers

##### Identification of Lignin- and Nylon 6-Specific Peaks

**Figure 4:**
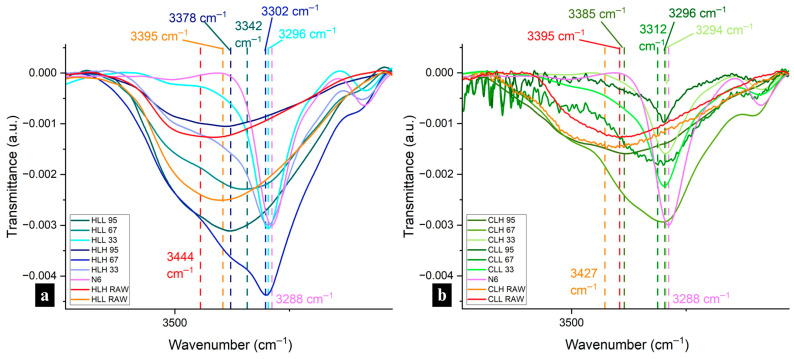
FTIR spectra of the OH region in nylon 6 fibers and (**a**) HL raw materials and HL-containing nanofibers, (**b**) CL raw materials and CL-containing nanofibers.

FTIR spectra were collected for the raw materials of lignin and nylon 6 for comparison with the lignin/nylon 6 nanofiber spectra (Figure S9a,b). For the lignin raw materials, lignin-specific peaks observed included a broad band in the OH region at 3400 cm^−1^, bands at ~1701 cm^−1^ due to carbonyl–carboxyl stretching, aromatic skeleton vibrations at around 1603 (S) and 1514–1513 (G), 1428–1423 (aromatic ring stretching with in-plane C–H deformation), 1329 cm^−1^ and the 1265 cm^−1^ peaks representing the C–O stretching of the S ring and G ring, respectively. Peaks at 1329 cm^−1^ were especially pronounced in the hemp hurd lignins. In corn stover lignin only, 1163 cm^−1^ peaks (C=O stretching in the ester group of HGS lignin) and 743 cm^−1^ peaks for C–H out-of-plane bending at positions 2, 5, and 6 of the aromatic ring in the H unit were also observed. Aligned with ^31^P NMR highest total OH analysis, HLL exhibited the broadest peak in the hydroxyl region. The OH region bands for the other fractions were similar. The neat nylon 6 sample was characterized by a N–H stretching vibration peak at 3288 cm^−1^ (Figure 4a,b), an amide I carbonyl peak at 1640 cm^−1^, a very weak amide II N–H deformation peak at 1535 cm^−1^ and an amide III band in the 1366–1257 cm^−1^ region (Figure S10a,b).

##### Hydrogen Bonding Interactions Between Lignin and Nylon 6

Spectra were collected for all electrospun lignin/nylon 6 samples (Figure S9) and analyzed for evidence of interactions between the two components. Figure 4a,b shows the region including the broad lignin OH band and sharper nylon 6 N–H stretching (3288 cm^−1^) and was used to assess hydrogen bonding between the two components. The shifts in the ~3500 cm^−1^ hydroxyl/3288 cm^−1^ N–H stretching region reflect both lignin–lignin and lignin–nylon 6 hydrogen bonding interactions. Across samples with lignin at all concentrations and from all biomass origins, CLL and HLH experienced the most significant downward shifts, indicating strong interactions with nylon 6.

The nylon 6 N–H stretching peak was present, sharp and unshifted in all 33:67 lignin/nylon 6 samples, pointing to strong interactions between lignin and the amide groups in nylon 6 (Figure 4a,b). Simultaneously, significant shifts from the initial 3426–3400 cm^−1^ OH peaks in raw lignin to the 3301–3298 cm^−1^ N–H stretching region indicate strong lignin-nylon 6 interactions (Figure 4a,b). In HLH, CLL and CLH 67, the N–H stretching peak, while not sharp, was present and exhibited broadening. These three samples also showed a high degree of shifts (−100 to −123 cm^−1^) in that region, which further confirms strong hydrogen bonding interactions with nylon 6. While broadening was also present in HLL 67, it experienced the lowest downward shift of −57 cm^−1^, pointing to decreased interactions with nylon 6 (Figure 4a). Fibers with 95:5 lignin:nylon 6 composition showed broad bands in the OH region, resembling raw lignin spectra, most likely explained by the very low nylon 6 content (Figure 4a,b). CLL 95 was an exception showing significant peak broadening and a significant redshift to 3297 cm^−1^ (N–H stretching), pointing to the strongest hydrogen bonding interaction with nylon amide groups among these samples. Between high- and low-M_w_ samples of each biomass origin, 95% samples containing higher-M_w_ lignins experienced only a minor downward shift of −66 to −42 cm^−1^, indicating diminished hydrogen bonding with nylon 6.

Shifts in the amide I region (carbonyl peak) to lower wavenumbers and broadening indicate weakening of the C=O bond and the presence of hydrogen bonding interactions between the hydroxyl groups in lignin and the carbonyl groups in nylon 6, which reconfirms the intermolecular interactions observed in the OH region. Weak downward shifts were present in all samples, but they were less significant in samples with high-M_w_ lignin fractions, pointing to decreased hydrogen bonding with nylon 6 (Figures S10a,b and S11a,b). HLH and CLH 95 showed no peak in that region which is most likely due to the low nylon 6 content and the overlap of aromatic ring vibrations in that region resulting from the high lignin content. Despite a sharp downward shift in the OH region, no amide I shift or change in intensity was observed in HLL 33, suggesting an absence of interaction with nylon 6 amide groups due to the low concentration of lignin in the sample and in line with the reduced interactions with nylon 6 for the HLL-containing samples observed in the OH region.

Similarly, amide II band (N–H bending) can be used as secondary verification of lignin–nylon 6 interactions in the fibers. While already very weak in neat nylon 6, this peak showed a downward shift to 1533 cm^−1^ in HLL 33, which previously did not show any interactions with nylon 6 in the amide I region; this serves as only a weak validation of the presence of any interactions (Figure S10a,b). The other two upward shifts observed in that region at 1549 cm^−1^ and 1544 cm^−1^ for CLL 67 and CLL 95 signify the weakening of the amide group bonds in nylon 6. Similarly to the amide I region, samples containing high-M_w_ lignin fractions showed no peak presence, confirming a lack of significant interactions with nylon 6.

##### Hydroxyl Group Accessibility in Lignin/Nylon 6 Composite Fibers

**Figure 5:**
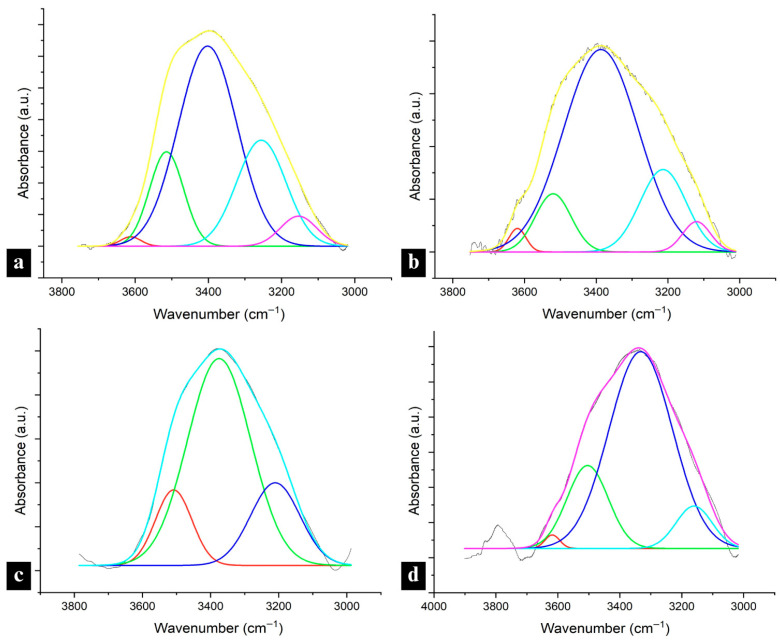
Deconvoluted FTIR spectra of the OH region in high M_w_ lignin raw materials: (**a**) HLH, and (**b**) CLH; and in 95:5 lignin:nylon 6 nanofibers containing (**c**) HLL, and (**d**) CLH fractions. The colored curves denote individual spectral components obtained through peak deconvolution of the OH stretching region.

While the presence and extent of lignin–lignin and lignin–nylon 6 hydrogen bonding can justify the variability of some performance attributes in lignin–nylon 6 composite fibers, functionalities, such as UV-blocking, antioxidant performance and water repellency, can also be explained by the accessibility of the OH groups in lignin after blending into a composite network. Generally, the OH region of 3700–3500 cm^−1^ has been used to identify free OH groups [37], while in lignin, wavenumbers above 3600 cm^−1^ (3639–3616) were specified as representative of free hydroxyl groups [38]. Deconvolutions of the OH region showed a small presence of non-hydrogen-bonded OH groups (relative peak area of 0.05–0.08 absorbance·cm^−1^) only in high-M_w_ neat lignin fractions of both biomass origins, observed in the 3656–3610 cm^−1^ region (Figure 5a,b).

The spectra in all lignin raw materials also showed the dimeric formation of an intermolecular bond in the 3515–3508 cm^−1^ region. The lower end of that range was observed in corn stover lignin, pointing to a higher potential for stronger intermolecular hydrogen bonding compared with hemp hurd lignin [38].

In lignin/nylon 6 fibers, free hydroxyl-group peaks were not observed in any of the 33% or 67% samples, reconfirming strong lignin–nylon 6 hydrogen bonding at those concentrations. However, peaks corresponding to intramolecular hydrogen bonds in phenolic groups were detected in 67 corn stover-lignin-containing fibers 3588–3523 cm^−1^, and were especially pronounced in the CLL fraction (relative peak area: 1.27 vs. 0.92 in CLH), which corresponds with the larger phenolic OH content for CLL in ^31^P NMR (Table S1).

In fibers with the highest lignin content, CLH was the only sample that displayed small peaks associated with free OH groups at ~3610 cm^−1^ (in two out of three measurements). While the relative integrated peak area of 0.08 (absorbance·cm^−1^) remained unchanged from the CLH raw material, it experienced a downward shift from 3656 to 3610 cm^−1^, within the free OH region, pointing to the onset of interactions with the polar nylon 6 in the composite. CLH and HLL exhibited peaks in the dimeric intermolecular H-bonded OH region at 3511 and 3509 cm^−1^, respectively, indicating the presence of lignin–lignin hydrogen bonding (Figure 5c,d). These peaks were absent in the CLL and HLH 95, suggesting reduced lignin–lignin interactions and enhanced interactions with nylon 6.

Analysis of the OH, amide I, and amide II regions demonstrated variability in hydrogen bonding across lignin fractions and concentrations, with CLL consistently showing the strongest interactions with nylon 6 across all regions. Downward shifts in those regions and the absence of free-hydroxyl peaks confirm the significant lignin-OH-amide hydrogen bonding in lower- and medium-loading samples, while weaker or absent shifts, such as in HLL, high-M_w_ lignin samples or high-lignin loadings, point to reduced interactions with nylon 6. These findings show that while the largest OH content in lignin enhances hydrogen bonding interactions in blends, it does not guarantee performance due to interference from other factors, such as lignin purity. HLL, which had the lowest purity of all lignin fractions, showed the second-weakest interaction with nylon 6 despite having the highest total OH content. Additionally, while strong redshifts in the OH region and the absence of lignin–lignin hydrogen bonding for CLL and HLH indicate significant hydrogen bonding with nylon 6, the absence of amide I and II redshifts in the high M_w_ samples, including those with HLH, indicates the nylon 6 internal hydrogen bond network remained largely undisturbed in those samples. Furthermore, CLH 95 was the only sample with an identified free OH presence, consistent with the weakest hydrogen bonding interactions with nylon 6.

#### 3.3.3. Thermal Stability of Lignin/Nylon 6 Nanofibers

**Figure 6:**
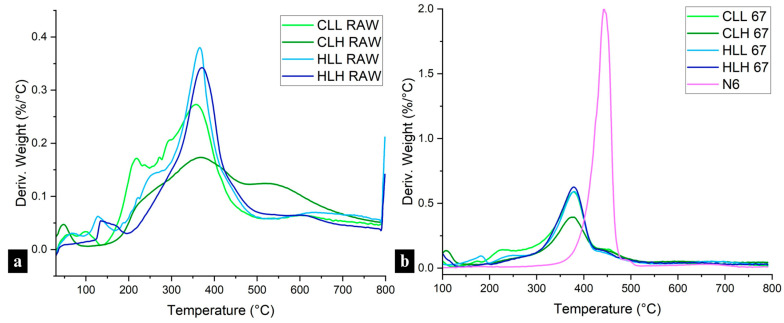
DTG curves of (**a**) all lignin raw materials, and (**b**) 67:33 lignin:nylon 6 and neat nylon 6 fibers from all lignin fractions.

TGA measurements were conducted to further confirm the interactions between nylon 6 and lignin. Neat nylon 6 fibers displayed a sharp DTG peak with an onset of degradation at 381 °C and a weight loss of 83.49% (Figure 6b). Overall, lignin addition decreased the onset temperature of degradation, but it also slowed the decomposition rate and increased the residual char from 2% in nylon 6 to 22–26% in 95:5 samples (Figure 6b and Figure S12a,b,d–f).

Multiple parameters showed a correlation with the thermal properties in lignin/nylon 6 samples. Higher total OH content (*r* = −0.36), linkage content (both, C–C, *r* = −0.52; and C–O bonds; *r* = −0.35), molecular weight (*r* = −0.53), and polydispersity (*r* = −0.54) contributed to lower total weight loss in the fibers (*p* < 0.0001).

Across all concentrations, fiber samples with hemp-hurd-lignin showed a more rapid decomposition compared to those with corn-stover-lignin, which is consistent with the observations of sharp DTG peaks in the hemp hurd lignin raw material (Figure 6a and Figure S12c). This is in line with the structural linearity of hemp hurd lignin, as evidenced by its high S/G ratio, which points to a more homogenous structure and easier decomposition (Figure 1b; Table S2). An opposite trend was observed in corn stover lignin, whose broader DTG peaks (Figure 6a,b and Figure S12a,b) reflect its more heterogenous structure, confirmed by a lower S/G ratio and the presence of H-units as discussed in the structural analysis in Section 3.3.2. Neat hemp hurd lignin also showed a higher β–O–4 linkage content and a lower condensation ratio (Table S2), making it more susceptible to degradation, which affected hemp hurd lignin/nylon 6 composite fiber thermal performance. However, while the presence of C–C bonds was lower than C–O bonds in hemp hurd lignin, it was higher than in corn stover lignin, leading to slightly higher residual char.

In terms of lignin molecular weight, lignin/nylon 6 samples with high-M_w_ fractions showed less drastic 2nd (post-moisture-loss) decomposition onsets, in the 329–331 °C range for 67% samples (Figure 6b and Figure S12e), and greater char formation compared to that observed in samples with low-M_w_ fractions, consistent with the lower β–O–4 linkage content and a higher condensation ratio present in those fractions (Figure 1b; Table S2). Higher lignin loadings further amplified these behaviors.

The broad DTG curves of neat lignin (Figure 6a) compared to the narrow temperature interval decomposition of nylon 6 stem from the heterogeneous lignin structure, leading to complex multi-step degradation processes. The presence of broad merged peaks within a single continuous degradation profile observed in all lignin/nylon 6 fibers confirms the interaction between the two polymers. This is further supported by the lower degradation onset and increased residual char in the blended fibers when compared with neat nylon 6 fibers (Figure 6b and Figure S12a,b,d–f).

These findings suggest that higher-M_w_ lignin fractions are most suitable for use in flame-retardant protective textiles requiring greater char formation. In terms of biomass source, lignins with lower S/G ratios, originating from residues such as corn stover, are more desirable for textile applications where a slower rate of thermal degradation is needed.

#### 3.3.4. Mechanical Strength of Lignin/Nylon 6 Nanofiber Membranes

DMA measurements of all fiber samples showed time-dependent mechanical behavior, typical of viscoelastic materials, as expected. Overall, the presence of lignin in the blends resulted in a decrease in tensile strength, elongation at break, and an increase in Young’s modulus, with some exceptions (Table S3). During measurements, neat nylon 6 necked under tensile stress, while the lignin-containing samples remained intact widthwise, pointing to lignin interference in the orientation of nylon 6 in the direction of applied stress resulting in brittle fibers.

While differences stemming from lignin biomass origin were not as pronounced in mechanical properties as differences originating from lignin molecular weight, corn stover lignin/nylon 6 blends displayed slightly higher tensile strength and Young’s modulus compared with hemp hurd lignin/nylon 6 samples (Figure 3a; Table S3). Although higher loading low M_w_ lignin-containing samples were too brittle to be mounted on the instrument, an increase in stiffness and equivalent or increased tensile strength in 33% samples pointed to enhanced interactions between lignin and nylon 6. Relative to 100% nylon 6, CLL 33, which has a 67% composition of nylon 6, exhibited higher tensile strength, a higher Young’s modulus, and lower ductility, suggesting dimensional stability and a well-developed interphase between lignin and the nylon 6 polymer matrix. Samples containing high-M_w_ lignin fractions exhibited lower tensile strength and elongation and lower ductility compared to their low-M_w_ counterparts, which aligns with the poorer hydrogen bonding interactions for those samples discussed in Section 3.3.2. In particular, 33:67 high-M_w_ lignin:nylon 6 samples actually showed an increased Young’s modulus compared to neat nylon 6, pointing to the presence of some degree of interaction between the two polymers even in high-M_w_ lignin-containing fibers.

Aside from lignin molecular weight, the thermal properties of lignin/nylon 6 fibers also dictated their mechanical performance, with low-residual char samples showing improved tensile strength and modulus (*r* = −0.75 and *r* = −0.58; *p* < 0.0001). Correspondingly, the tensile strength, elongation at break and Young’s modulus of the nanofiber mats also showed a positive correlation with total TGA weight loss (r = 0.58, r = 0.50, r = 42; *p* < 0.0001), underlining the most optimal mechanical properties in a sample with the most effective lignin–nylon 6 hydrogen bonding network, CLL 33.

Overall, the tensile strength of the tested nanofiber membranes is lower compared with that of synthetic nanofiber membranes which have reported tensile strength of 10–12.5 MPa [39] and ~2–13 MPa [40] when used for protective textile applications. However, increasing the thickness of the lignin/nylon 6 membranes could help address these shortcomings in real-world applications.

#### 3.3.5. Antioxidant Activity of Fibers

**Figure 7:**
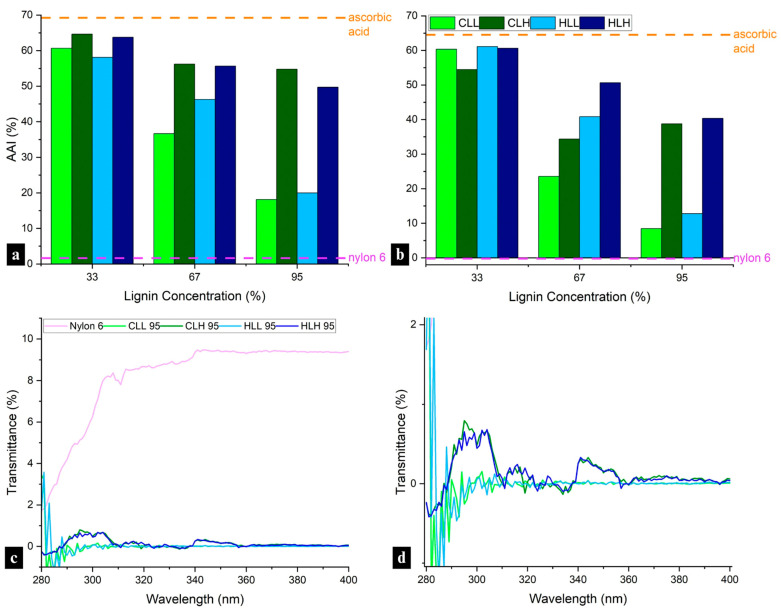
Antioxidant activity index of all nanofiber mats by lignin composition, with ascorbic acid as positive control and nylon 6 fibers as negative control, after (**a**) 10 min, and (**b**) 24 h of assay initiation. Bar colors correspond to lignin fraction present in the sample, with CLL, CLH, HLL, and HLH compositions indicated in the legend. (**c**) Comparison of UV transmittance of 95:5 lignin/nylon 6 to neat nylon 6 fibers, and (**d**) closeup of UV transmittance of all 95:5 lignin/nylon 6 fibers. Curve colors correspond to the different samples, as indicated in the legend.

Addition of lignin to nylon 6 blends significantly increased the antioxidant activity (AA) of the electrospun fiber mats, with AAI values ranging from 18 to 65% after 10 min and 8–61% after 24 h, approaching the performance of ascorbic acid and far exceeding that of neat nylon 6 (Figure 7a,b). While the inherent antioxidant activity of lignin has been mainly attributed to higher phenolic OH content [41,42], other factors must be considered in lignin blends with synthetic polymers, such as nylon 6. In such composite systems, AA is positively influenced by higher phenolic OH accessibility, S/G ratio, the presence of other functional groups such as C=C bonds, carbonyl groups, lignin solubility and polymer compatibility, and negatively influenced by higher total OH content (with a focus on aliphatic and carboxylic acid OH groups), and the presence of other functional groups such as -CHO, and -CO groups [43,44].

A slight decrease in AAI over time was observed for all samples (Figure 7b), but most notably, the AAI in samples containing both lignin fractions showed a strong negative correlation with lignin concentration (samples with low-M_w_ lignin: adjusted *R*^2^ = 0.94; *p* < 0.005; with high-M_w_ lignin: adjusted *R*^2^ = 0.87; *p* < 0.005). Simultaneously, across all samples, phenolic OH decreased with an increase in AAI, opposite to expectations (Figure S13a,b). A linear regression showed that this impact was significant both for fibers with high M_w_ (adjusted *R*^2^ = 0.90; *p* < 0.005) and low M_w_ lignin (adjusted *R*^2^ = 0.96; *p* < 0.005). The decrease in correlation between AAI and phenolic OH content across the lowest lignin concentration samples (*r* = −0.80 compared to *r* = −0.97–0.99 in higher lignin concentration samples, *p* < 0.01) suggests other underlying factors contributing to this effect. The improved antioxidant performance at the lowest lignin concentration in the composite fibers can be explained by the phenomenon of an optimal lignin concentration for radical scavenging, which has also been reported in other studies. Domínguez-Robles et al. (2020) observed similar initial antioxidant activity for lignin/poly(butylene succinate) composites with lignin concentrations 5% and 10% wt., and a lower AA for a sample with 15% wt [45]. Any increase in lignin concentration beyond the optimal concentration did not simultaneously increase antioxidant activity in the blends, which was also observed in this study.

Furthermore, in addition to variability in AAI at different lignin concentrations, the differences between samples containing the low- and high-M_w_ lignin fractions cannot be explained with phenolic OH content alone; they point to morphological differences, including aggregation mechanisms, that correlate with the higher lignin heterogeneity and the reduced lignin–lignin and lignin–nylon 6 intermolecular interactions in high-M_w_ fractions discussed in Section 3.3.2.

In terms of biomass origin, when compared with AA of HGS-type corn stover lignin, the AA of samples with hemp hurd lignin was slightly higher and positively influenced by the much higher S/G ratio (Table S2), which offset the lower phenolic OH content with additional stabilization through the dimethoxy structure.

To fully take advantage of antioxidant properties of lignin in filtration membranes, cosmetic care, outdoor and medical protective textiles, as well as composite textiles where it enhances the durability of synthetic polymers such as nylon 6, blends with lower concentrations of lignin that show improved phenolic OH accessibility for radical scavenging are recommended. While normally, low–M_w_ lignin fractions with higher phenolic OH contents are preferable for these applications, in blends with nylon 6, higher-M_w_ lignin fractions show superior performance due to their comparatively higher phenolic OH accessibility.

#### 3.3.6. UV Absorbance of Fiber Mats

To investigate potential use in outdoor protective materials, the UV-blocking performance of the lignin/nylon 6 nanofiber membranes was evaluated. Incorporating lignin increased the UPF rating of nylon 6 from 12 to 50+ for all composite mats. Low-M_w_ lignin fractions from both biomass origins delivered the highest protection, achieving exceptionally low UV diffuse transmittance (0.06–0.09%) at the highest lignin concentrations (Figure 7d). High-M_w_ lignin fractions showed significantly lower performance, with transmittance values in the range of 0.09–0.23%, though still markedly better than neat nylon 6 (8.6% transmittance; Figure 7d). This is consistent with the lower phenolic OH content in high M_w_ lignin fibers, as well as reduced hydrogen bonding with nylon 6. The strongest UV-blocking was observed in lignin-rich CLL 95 (0.08%) and HLL 95 (0.06%), which correlates with the abundant phenolic groups responsible for UV absorbance in low-M_w_ lignin (Figure 1a; Table S1). While the dense fibrous network of an electrospun nanofiber mat can provide some minor physical UV-blocking mechanisms, as demonstrated in the neat nylon 6 sample (Figure 7c), most UV blocking is activated after the addition of lignin to the composite fiber mats. These results confirm that lignin can inherently impart durable UPF 50+ performance without additional chemical finishing, resulting in UV blocking that is 100 times better than that of nylon 6 alone.

Using lower-M_w_ phenolic OH-rich lignin fractions will be most beneficial for achieving >99.9% UV absorbance, meeting the highest UPF 50+ standards applied in protective textiles used for outdoor workwear, Personal Protective Equipment (PPE), shades, and in outdoor filtration membranes, such as the ones used in agriculture or construction.

#### 3.3.7. Water Vapor Permeability of Nanofiber Membranes on Cotton and Nylon Fabric Substrates

Maintaining adequate water vapor permeability (WVP) is essential for comfort and microclimate regulation in protective apparel and for moisture management in packaging systems. A nanofiber membrane layer on a fabric substrate can serve as a functional protective barrier while maintaining breathability.

**Figure 8:**
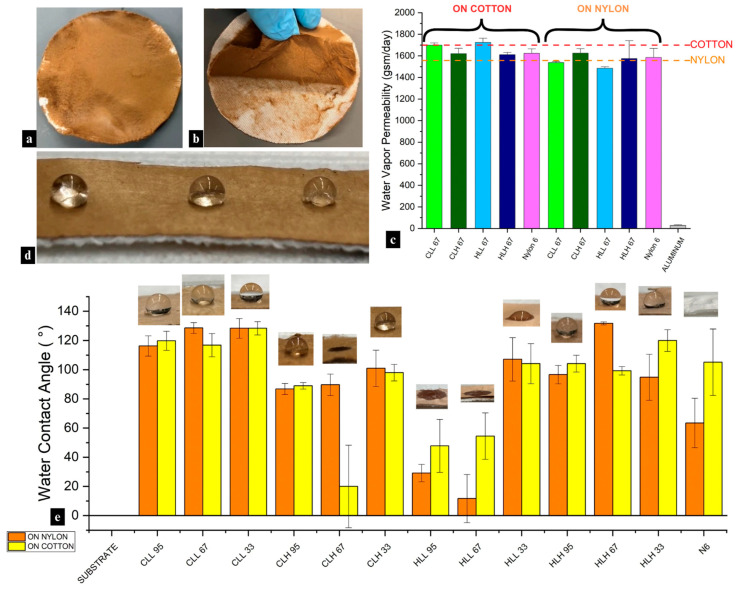
Images showing (**a**) lignin/nylon 6 nanofiber membrane-coated nylon textile, (**b**) peeled membrane due to poor adhesion. (**c**) Water vapor permeability of neat cotton substrate and neat nylon substrate, each followed by 95:5 lignin/nylon 6 and neat nylon 6 fiber samples, and aluminum foil as control. (**d**) Image of water droplet on surface of lignin/nylon 6 nanofiber membrane-coated nylon textile and (**e**) average water contact angle measurements with corresponding images of water contact angle droplet on all samples.

Electrospinning lignin/nylon-6 nanofiber membranes onto cotton did not alter the fabric’s inherent breathability as the composite structures preserved 92–101% of the original water vapor permeability of the substrate (Figure 8c and Figure S14a,b). As expected, hydrophilic neat cotton exhibited higher WVP (1708 gsm/day) than hydrophobic neat nylon (1557 gsm/day). Interestingly, when lignin/nylon-6 nanofiber membranes were deposited onto nylon (Figure 8a), some composites showed WVP values above those of the original substrate (see CLH 95; Figure S14b), reaching 107%, indicating that the nanofiber layer can, in some cases, facilitate rather than restrict vapor transmission.

The influence of nanofiber membrane composition varied by substrate (*p* < 0.05), but nanofiber mat mass (*r* = 0.03; *p* < 0.001), thickness (*r* = 0.5; *p* < 0.001), or pore size (*r* = 0.02–0.11; *p* < 0.001) did not correlate with permeability. The observed patterns cannot be attributed to chemical interactions between the membrane and substrate due to a lack of adhesion (Figure 8b) resulting from high solvent volatility during electrospinning. Instead, variability was due to the differences in through-thickness morphology and porosity of the nanofiber layer, physical interactions with the substrate, and the polarity and surface functional groups of both layers, which regulate sorption–desorption. Additionally, the observed fiber morphologies between samples containing different lignin fractions did not drastically differ from one another (Figure S15a–d), and fiber diameter showed only a moderate correlation (*r* = 0.49 on nylon; *r* = 0.28 on cotton; *p* < 0.001).

Lignin hydroxyl content had a significant impact on the WVP performance of the composite membranes on both substrates (*p* < 0.001). While variability in WVP between low- and high- M_w_ lignin/nylon 6 membranes on the polar cotton substrate was not significant, it differed on nylon (*p* < 0.001). In the latter, high-M_w_-lignin membranes with lower hydroxyl content and higher proportions of aromatic linkages, such as β-O-4, β-β, β-5 (Figure 1a,b; Tables S1 and S2), yielded higher WVP (1500–1666 gsm/day) due to their reduced surface polarity, eliminating interfacial moisture uptake, and facilitating vapor transmission through the nanofiber layer pores.

Increasing nylon 6 concentration in the blends generally enhanced WVP (Figure S14a,b) by increasing the accessible amide groups, which slightly improved the rate at which water vapor entered and exited the membrane surface. Membranes containing HLH fractions were an exception, showing an increase in WVP on cotton with higher lignin content (Figure S14a). It is likely that the combination of the reduced polarity of higher-lignin membranes and the largest average pore size (~13 μm) both contributed to the improved performance. In contrast, in membranes with low M_w_ marked by higher OH content, decreasing lignin content increased WVP on both substrates (Figure S14a,b).

#### 3.3.8. Water Repellency of the Fiber Mats and Fiber Membranes on Cotton and Nylon Substrates

Lignin-loaded breathable composite nanofiber membranes can also offer inherent and durable water repellency on the surface through unique lignin chemistry, fiber morphology, and interactions with the substrate, increasing the contact angle from 0° on the substrate to >90°. While quickly absorbed, the presence of minor initial moisture repellency in neat nylon 6 membranes pointed to the influence of the nanofiber network’s morphology on wetting behavior (Figure 8e). Additionally, a lack of uniformity throughout the fiber mats across samples contributed to the differences in water repellency between samples as the micro/nanostructure surface roughness can amplify both hydrophilic and hydrophobic behavior.

Neat nylon 6 itself exhibited moderate initial hydrophobicity but absorbed water and deformed quickly, while in most cases, the incorporation of lignin in lignin/nylon 6 nanofiber membranes improved hydrophobic performance and dimensional stability to water relative to neat nylon 6 (Figure 8d). There were exceptions (Table S4), which emphasized the fact that the sole lignin presence does not guarantee hydrophobicity. These differences, similar to water vapor permeability, were mainly driven by lignin–OH bonding mechanisms. Samples containing HLH and CLL lignin consistently performed better in terms of surface water repellency compared to CLH and HLL. This behavior was not dictated by lignin M_w_ nor its biomass origin. Instead, CLL and HLH exhibited the strongest hydrogen-bonding interactions with nylon 6, as evidenced by strong downward shifts in the hydroxyl and amide I region and the absence of key lignin–lignin OH bonding peaks observed in samples containing the other fractions. Additionally, the lower total hydroxyl content of HLH, along with a higher M_w_, reduced its polarity within the composite and limited interactions with water, resulting in long-term water resistance.

Samples containing CLH and HLL consistently displayed lower contact angles or rapid wetting. This was especially pronounced in HLL-containing stand-alone nanofiber membranes, which showed reduced water repellency ranging from 45 to 95° and, in most cases, approached complete wetting within seconds (Table S4). These behaviors correspond directly to the lowest lignin purity in HLL compared to other lignin fractions, signifying the presence of carbohydrate impurities that could increase the polarity of the composite fibers (Table 2). While CLH had a higher M_w_ and the lowest total OH content compared to other lignins, the lack of hydrogen-bonding shifts for this sample in the hydroxyl region points to enhanced hydrophilicity in the membrane.

The performance trends observed on neat nanofiber membranes were largely mirrored when the membranes were electrospun onto nylon and cotton substrates (Figure 8e; Table S4). On both highly hydrophilic cotton and less hydrophilic nylon substrates, the deposition of lignin/nylon 6 membranes largely suppressed any hydrophilic behavior. As expected, CLL and HLH membranes deposited on both substrates exhibited durable water repellency. Overall, while certain samples showed increased variability in water contact angle (WCA) values across the two substrates, evidenced by larger standard deviations, WCA values for most samples were comparable across the two substrates. These findings highlight that, in addition to fiber architecture and blend composition, deliberate lignin fraction selection can permit control over water repellency in lignin-containing fiber systems by tailoring structural characteristics rather than relying on lignin presence alone.

These nanofiber membranes are especially recommended for use in PFAS (per- and polyfluoroalkyl)-free durable, non-leaching, waterproof, breathable protective textiles for outdoor workwear and PPE, filtration, as well as in medical and cosmetic textiles. While biomass origin and fractionation can help tailor water-repellent properties in lignin/nylon 6 nanofiber membranes, the selection of the most suitable lignin is guided by highest purity and strongest lignin–polymer OH bonding, which in this study was observed in the CLL and HLH fractions.

## 4. Conclusions

This study demonstrates the effective valorization of fractionated CELF lignins from agricultural residues, hemp hurd and corn stover, into multifunctional lignin/nylon 6 smooth nanofiber membranes, with lignin M_w_ and botanical origin serving as the key determinants of lignin structure, impacting interactions with nylon 6 and composite performance. Overall, the addition of lignin improved UV protection, antioxidant activity, and, in most cases, water repellency across all fractions.

The variations in chemical composition in lignin fractions, including OH and linkage content, as well as S/G and condensation ratios, directly affected the functional performance in fiber membranes regardless of biomass origin. Higher lignin purity contributed to better water repellency and enhanced interactions with nylon 6. Higher phenolic OH content increased UV blocking and contributed to antioxidant properties. Lignin/nylon 6 nanofibers containing lower-M_w_ lignin fractions showed the most optimal UV-protection, smaller-diameter, brittle fibers and the strongest OH bonding with nylon 6, while those with higher-M_w_ lignin fractions showed better thermal properties. While higher lignin concentrations increased UV absorbance, they simultaneously decreased mechanical strength, antioxidant activity, and OH bonding with nylon 6.

The extent and type (lignin–nylon 6 and lignin–lignin) of hydrogen bonding, as well as the presence of free OH groups governed lignin–nylon 6 interactions. These interactions, influenced by lignin OH content, molecular weight, and purity, simultaneously enhanced or suppressed the functional performance of the composite fibers. Accessibility of OH groups, such as the free OH in CLH 95, decreased hydrogen-bonding interactions with nylon 6, but increased antioxidant activity. At the same time, CLL samples showed strong lignin–nylon 6 interactions, contributing to greater UV absorbance and water repellency. The lowest lignin purity in HLL resulted in decreased hydrogen-bonding interactions with nylon 6, despite the highest OH content among the lignin fractions. Hence, while the magnitude of OH composition was critical in influencing lignin–nylon 6 interactions in blends, it competed with other factors, such as molecular weight and purity. Overall, controlling lignin–nylon 6 hydrogen bonding through lignin fraction selection enables the tailoring of nanofiber functional properties beyond lignin chemical composition alone.

When deposited onto textile substrates, lignin–nylon 6 nanofiber membranes preserved or even improved water vapor permeability while imparting inherent, long-lasting antioxidant activity, controlled PFAS-free water repellency, and UV protection 100 times better than that of nylon 6 alone, without the need for chemical finishing. As a result, this study positions lignin/nylon 6 nanofiber membranes as promising candidates for sustainable protective textiles, breathable water-repellent coatings, UV-shielding filtration layers, and photodegradation-resistant packaging.

Recommendations for future work include an expanded study using greater feedstock diversity, which would support further standardization of CELF lignin for scalable fiber processing, optimization of lignin fractionation using solvents that directly influence lignin miscibility with selected polymer, a study on the comparison of hydrogen-bonding and OH-accessibility mechanisms in composite lignin systems using different polymers, as well as a large-scale multivariate analysis of resulting structure–property relationships. Finally, testing the performance of lignin/nylon 6 nanofiber membranes in a select protective textile application according to industry standards would help accelerate their adoption for real-world applications.

## Figures and Tables

**Table 1:** Nanofiber mat samples with correlated lignin/nylon 6 composition, lignin fraction and sample labels.

Sample Label	CLL 95	CLL 67	CLL 33	CLH 95	CLH 67	CLH 33	HLL 95	HLL 67	HLL 33	HLH 95	HLH 67	HLH 33	N6
Lignin:Nylon 6 Ratio	95:5	2:1	1:2	95:5	2:1	1:2	95:5	2:1	1:2	95:5	2:1	1:2	0:100
Lignin Biomass Origin	Corn Stover						Hemp Hurd						N/A
Lignin Fraction	Low-M_w_ (Soluble)			High-M_w_ (Insoluble)			Low-M_w_ (Soluble)			High-M_w_ (Insoluble)			N/A

**Table 2:** Chemical composition and molecular weight distribution of CELF hemp hurd and corn stover lignins.

Lignin Fractions	Chemical Composition [%]		Molecular Weight Distribution	
	Lignin	Ash	M_w_ [g/mol]	*Ɖ*
CLL	96.5 ± 1.3	0.1 ± 0.0	1325 ± 128	1.7 ± 0.1
CLH	93.2 ± 1.3	1.1 ± 0.3	4623 ± 139	2.1 ± 0.1
HLL	87.6 ± 0.5	0.3 ± 0.3	3401 ± 45	1.6 ± 0.2
HLH	95.1 ± 0.1	0.0 ± 0.0	8040 ± 846	2.5 ± 0.3

## Data Availability

The raw data supporting the conclusions of this article will be made available by the authors on request.

## Supplementary Materials

The following supporting information can be downloaded at: https://www.mdpi.com/article/10.3390/polym18091124/s1, Table S1: Hydroxyl group content of CELF corn stover lignins (Unit: mmol/g). Table S2: Lignin subunit and linkage contents of CELF corn stover lignins. Table S3: DMA measurements for all samples. Samples with * were too brittle to be mounted and were not measured. Table S4: Average water contact angle measurements for all stand-alone nanofiber membranes. Figure S1: HSQC spectra of CLL and CLH. Figure S2: HSQC spectra of HLL and HLH. Figure S3: Corn stover lignin/THF solution-electrospun microfiber mat as shown on (a) a photograph after collection; and an optical micrograph at (b) 10× and (c) 4×. Figure S4: Differences in solubility between (a) CLH and (b) CLL fractions. Figure S5: (a) Illustration of lignin/nylon 6/HFIP solution electrospinning on a negative voltage setup, and (b) images of differences in electrospinning yield between (left: −) and (right: +) voltage for CLL 95 solution compared to neat nylon 6 (small corner image). Figure S6: SEM micrographs of lignin/nylon 6 samples along with their average fiber diameters. Figure S7: SEM micrographs portraying brittleness on (a,b) CLL 67 fibers and (c) HLL 67 fibers. Figure S8: (a,b) SEM micrographs portraying phase separation on HLH 33 fibers. Figure S9: FTIR spectra of nylon 6 nanofibers and (a) all HL-containing nanofibers, (b) all CL-containing nanofibers. Figure S10: FTIR spectra of amide regions in nylon 6 nanofibers and (a) all HL-containing nanofibers, (b) all CL-containing nanofibers. Figure S11: FTIR spectra of the C=O region in nylon 6 nanofibers and (a) all HL-containing nanofibers, (b) all CL-containing nanofibers. Figure S12: DTG curves of (a) 95% and (b) 33% lignin/nylon 6 fibers, and decomposition curves of (c) lignin raw material, (d) 95%, (e) 67%, and (c) 33% lignin/nylon 6 fibers. Figure S13: Linear regression plots of initial AAI and phenolic OH content in samples with (a) low M_w_ lignin, and (b) high M_w_ lignin. Figure S14: Water vapor permeability of neat substrate, aluminum foil as control, and lignin/nylon 6 and neat nylon 6 nanofiber membranes on (a) cotton fabric substrate, and (b) nylon fabric substrate. Figure S15: SEM micrographs of nanofiber samples collected from the nylon substrate: (a). CLL 2:1, (b). CLH 2:1, (c). HLL 2:1, (d). HLH 2:1.

## Abbreviations

The following abbreviations are used in this manuscript:

CL	Corn Stover Lignin
HL	Hemp Hurd Lignin
CLH	Corn Stover High M_w_ Lignin
CLL	Corn Stover Low M_w_ Lignin
HLH	Hemp Hurd High M_w_ Lignin
HLL	Hemp Hurd High M_w_ Lignin
AA	Antioxidant Activity
AAI	Antioxidant Activity Index
M_w_	Molecular Weight
