# Supplementary material for: Sustainable Protective Composite Textiles: Valorizing Hemp Hurd and Corn Stover Lignin via Electrospinning
Source: Polymers (Basel). 2026 May 2;18(9):1124. doi: 10.3390/polym18091124 (PMC13165889; doi:10.3390/polym18091124)
Supplement: Supplementary file 1 [file polymers-18-01124-s001.zip › polymers-4232952-supplementary.pdf]

Table S1. Hydroxyl group content of CELF corn stover lignins (Unit: mmol/g)

Lignin Fractions	Aliphatic OH	Aromatic OH	Carboxylic acid OH	Total OH
CLL	1.2 ± 0.1	3.2 ± 0.2	0.7 ± 0.1	5.2 ± 0.3
CLH	1.1 ± 0.1	2.3 ± 0.2	0.4 ± 0.1	3.9 ± 0.3
HLL	2.8 ± 0.0	2.9 ± 0.1	0.2 ± 0.0	5.8 ± 0.1
HLH	2.3 ± 0.0	2.4 ± 0.0	0.1 ± 0.0	4.8 ± 0.1

Table S2. Lignin subunit and linkage contents of CELF corn stover lignins

Lignin Fractions	S/G Ratio	Condensation Ratio	pCA [%]	FA [%]	β–O–4 [%]	β–β [%]	β–5 [%]
CLL	0.5	1.25	24.4	10.2	5.3	0.3	0.8
CLH	0.5	0.92	30.0	4.7	11.2	0.3	1.1
HLL	2.3	0.17	9.5	0.0	13.8	6.0	4.8
HLH	1.2	0.15	5.6	0.0	22.9	9.8	7.9

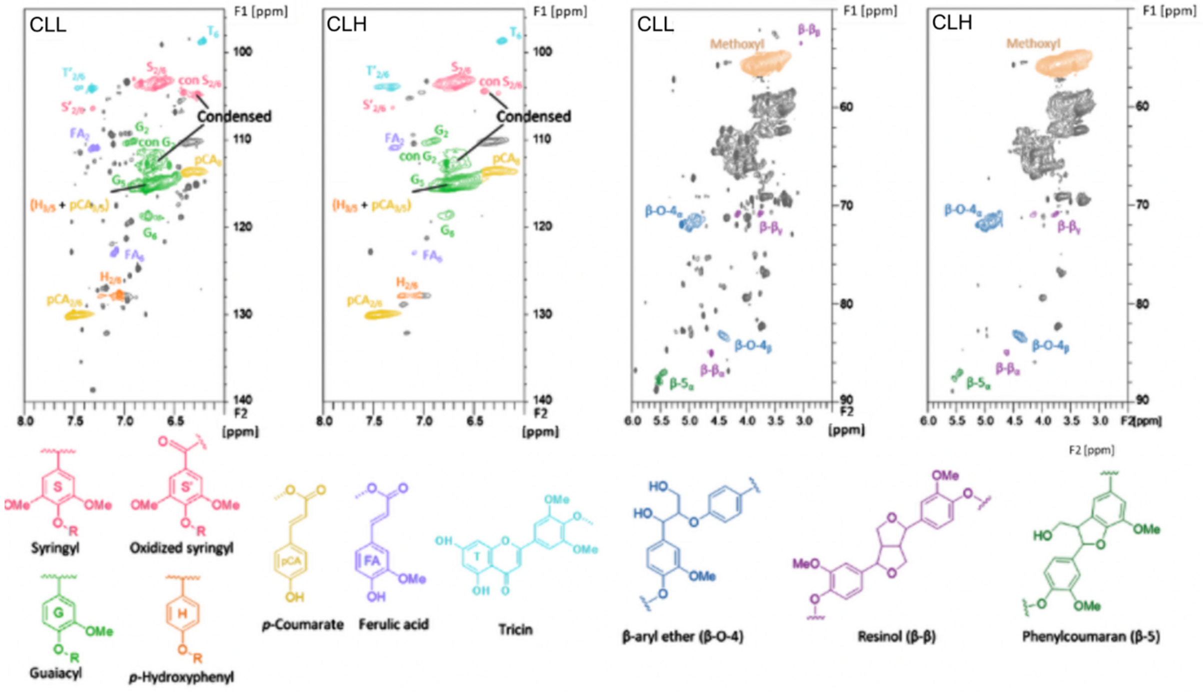

Figure S1. HSQC spectra of CLL and CLH.

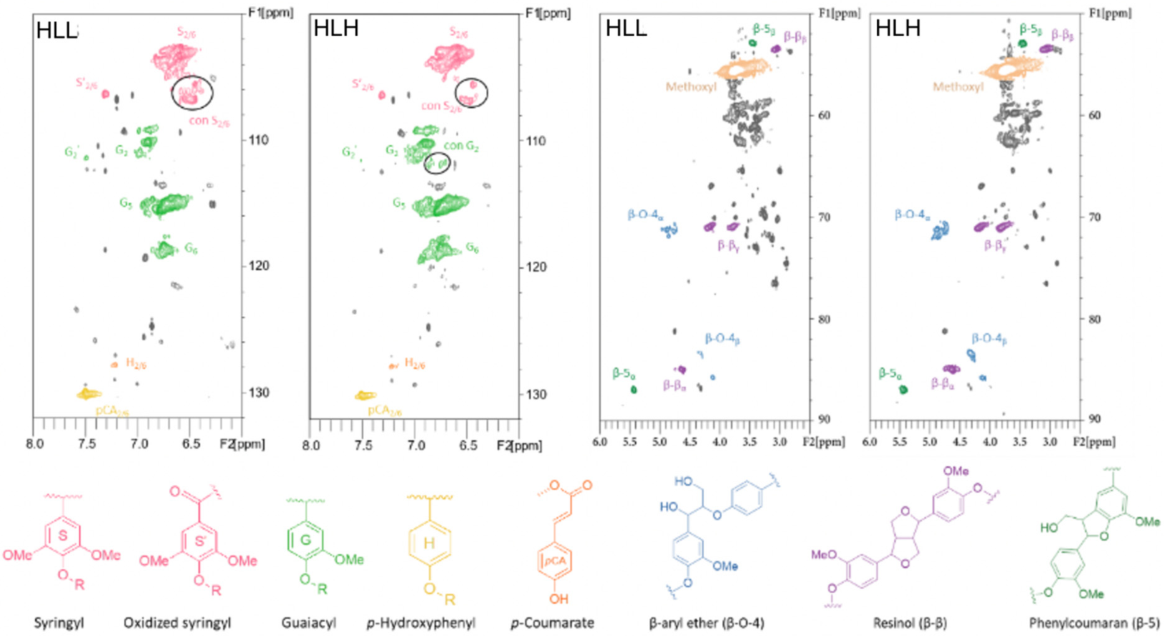

Figure S2. HSQC spectra of HLL and HLH.

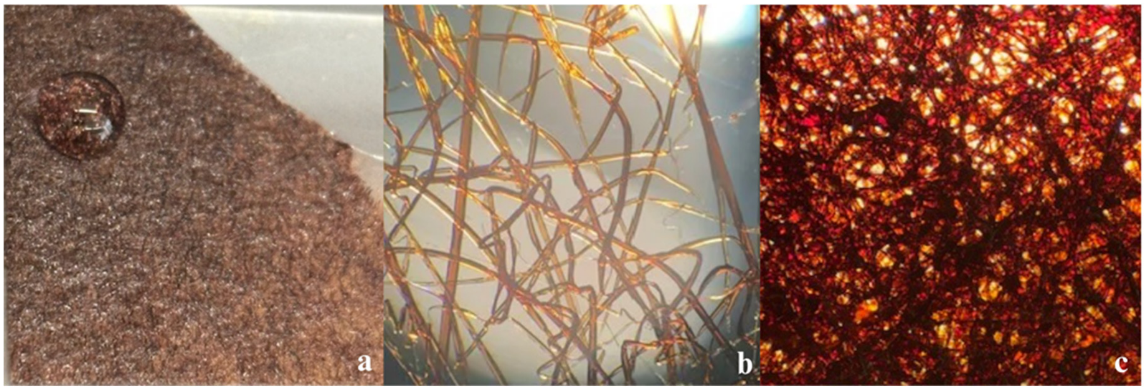

Figure S3. Corn stover lignin/THF solution-electrospun microfiber mat as shown on (a) a photograph after collection; and an optical micrograph at (b) 10x and (c) 4x.

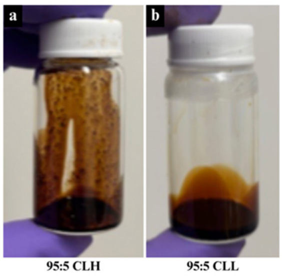

Figure S4. Differences in solubility between (a) CLH and (b) CLL fractions.

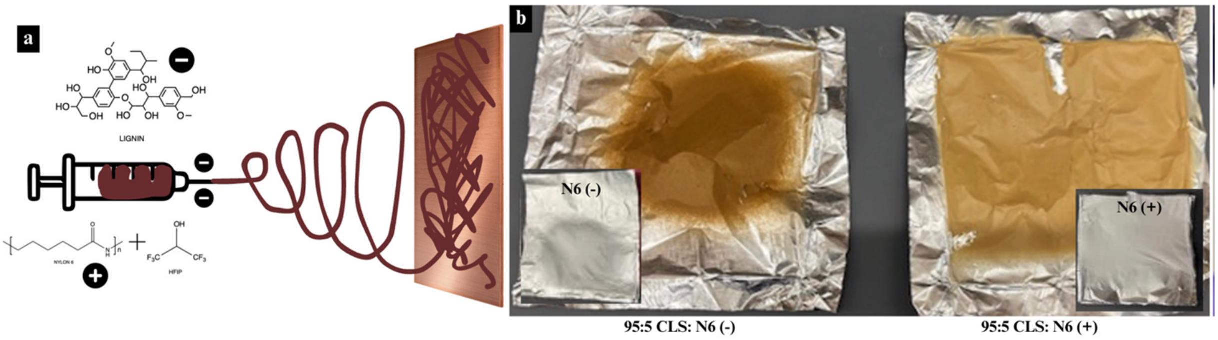

Figure S5: (a) Illustration of lignin/nylon 6/HFIP solution electrospinning on a negative voltage setup, and (b) images of differences in electrospinning yield between (left: -) and (right: +) voltage for CLL 95 solution compared to neat nylon 6 (small corner image).

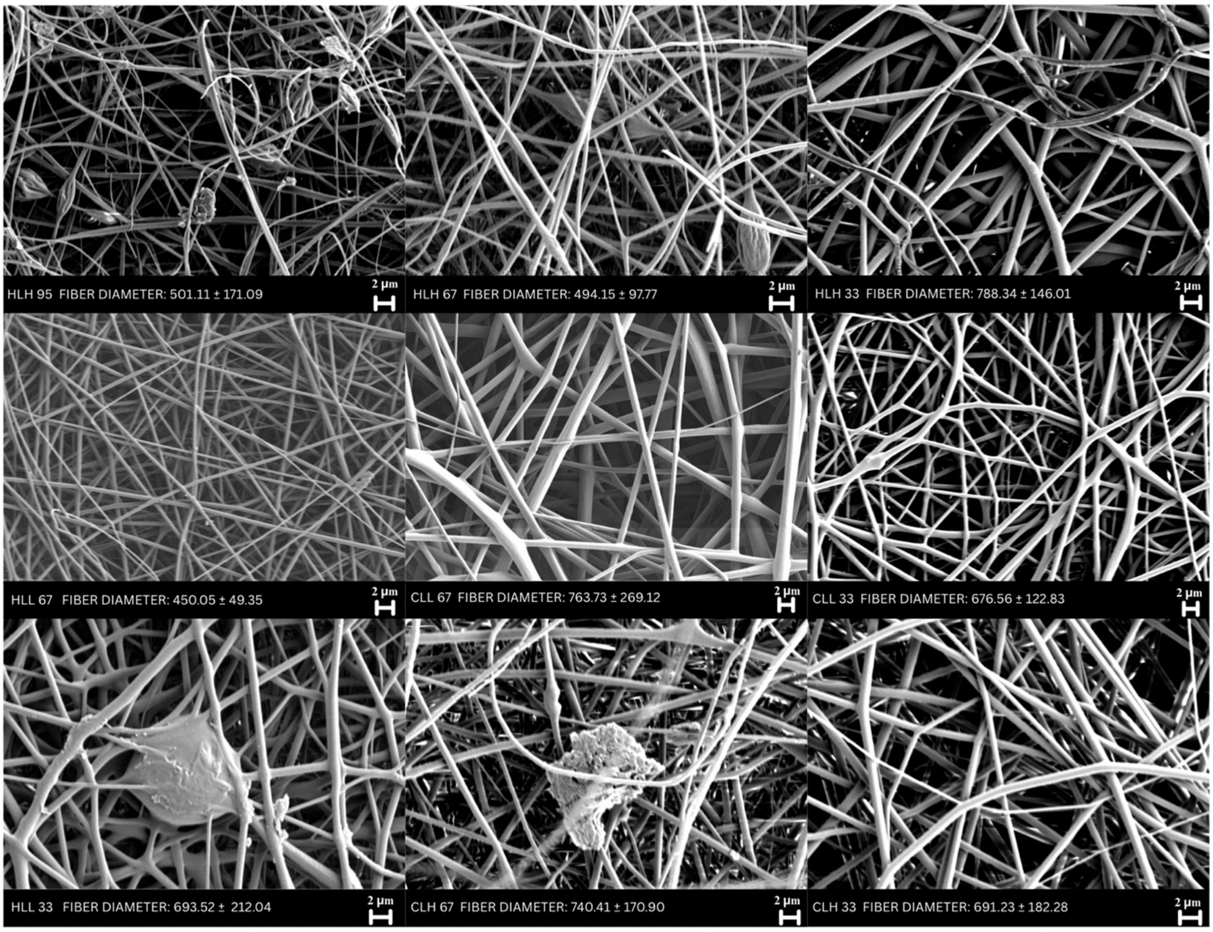

Figure S6. SEM micrographs of lignin/nylon 6 samples along with their average fiber diameters.

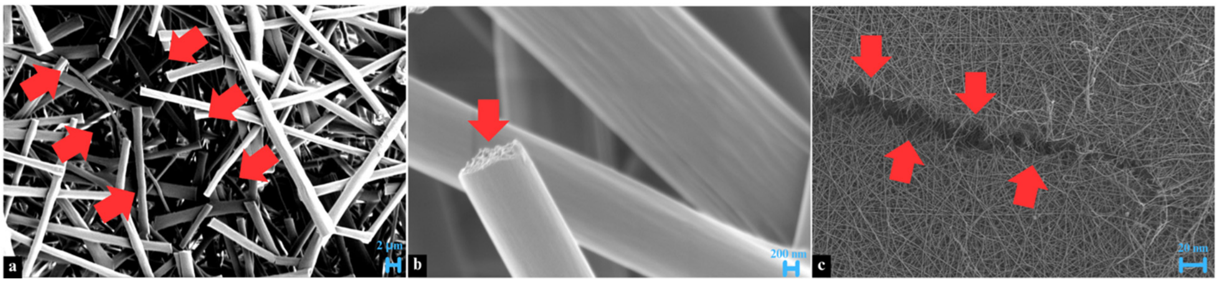

Figure S7. SEM micrographs portraying brittleness on (a-b) CLL 67 fibers and (c) HLL 67 fibers.

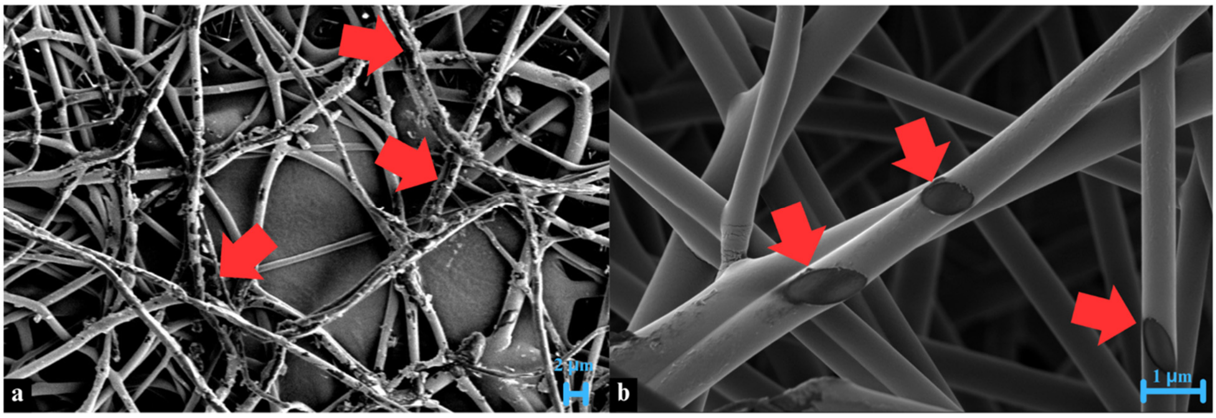

Figure S8. (a-b) SEM micrographs portraying phase separation on HLH 33 fibers.

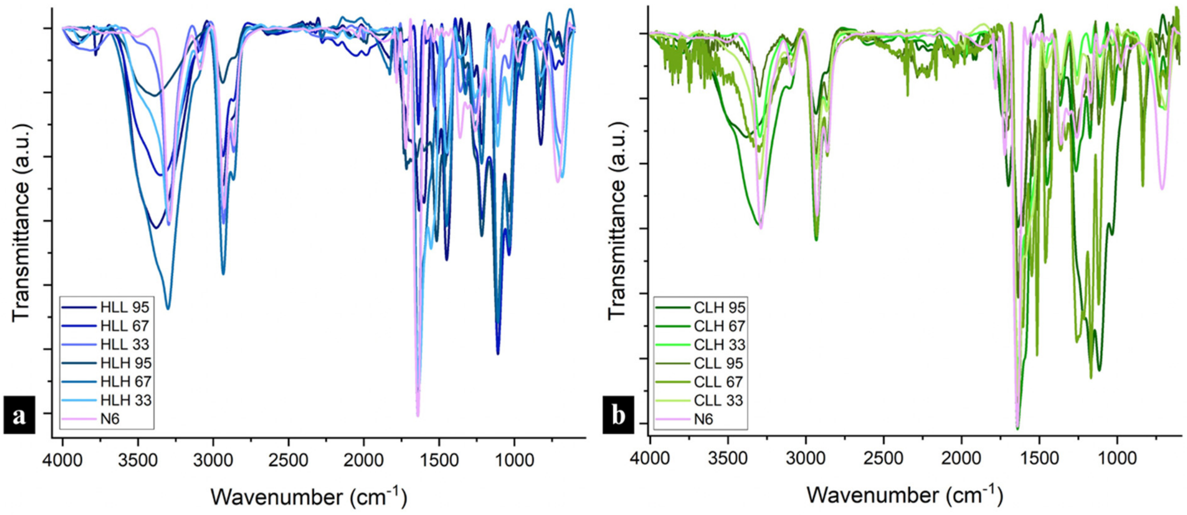

Figure S9. FTIR spectra of nylon 6 nanofibers and (a) all HL-containing nanofibers, (b) all CL-containing nanofibers.

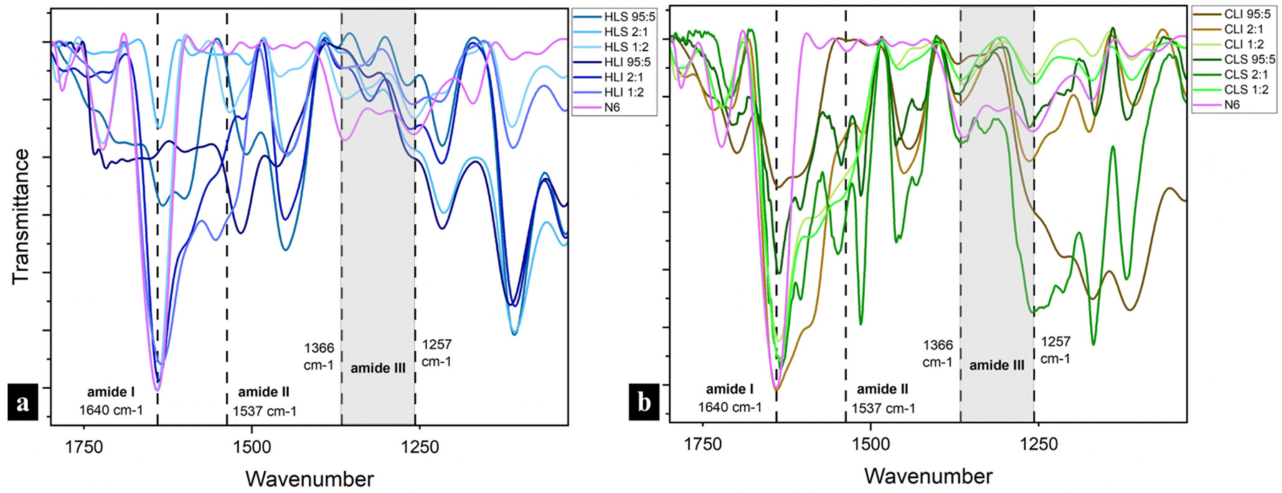

Figure S10. FTIR spectra of amide regions in nylon 6 nanofibers and (a) all HL-containing nanofibers, (b) all CL-containing nanofibers.

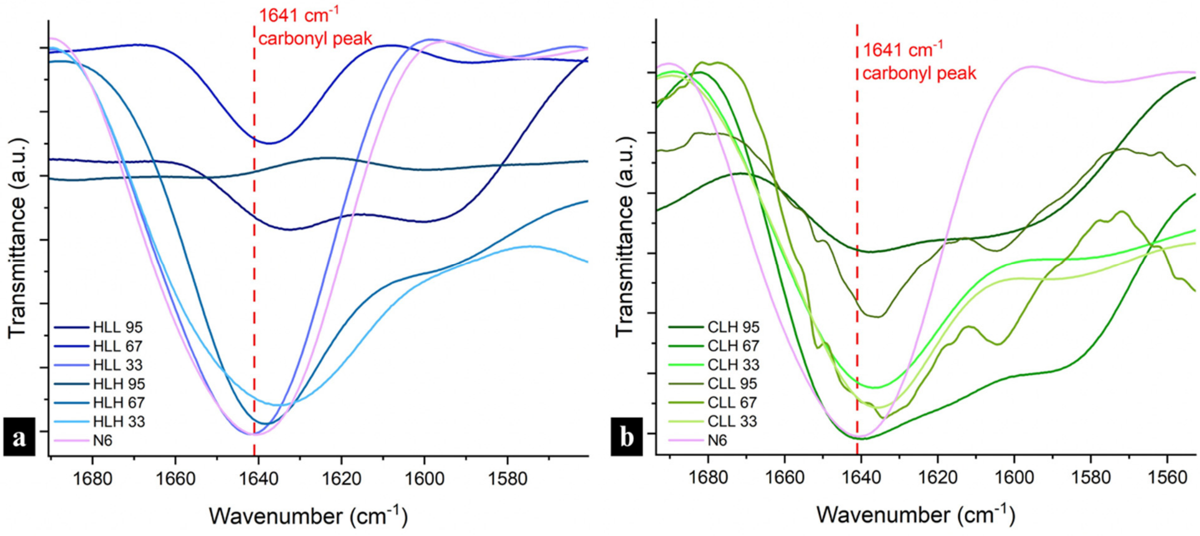

Figure S11. FTIR spectra of the C=O region in nylon 6 nanofibers and (a) all HL-containing nanofibers, (b) all CL-containing nanofibers.

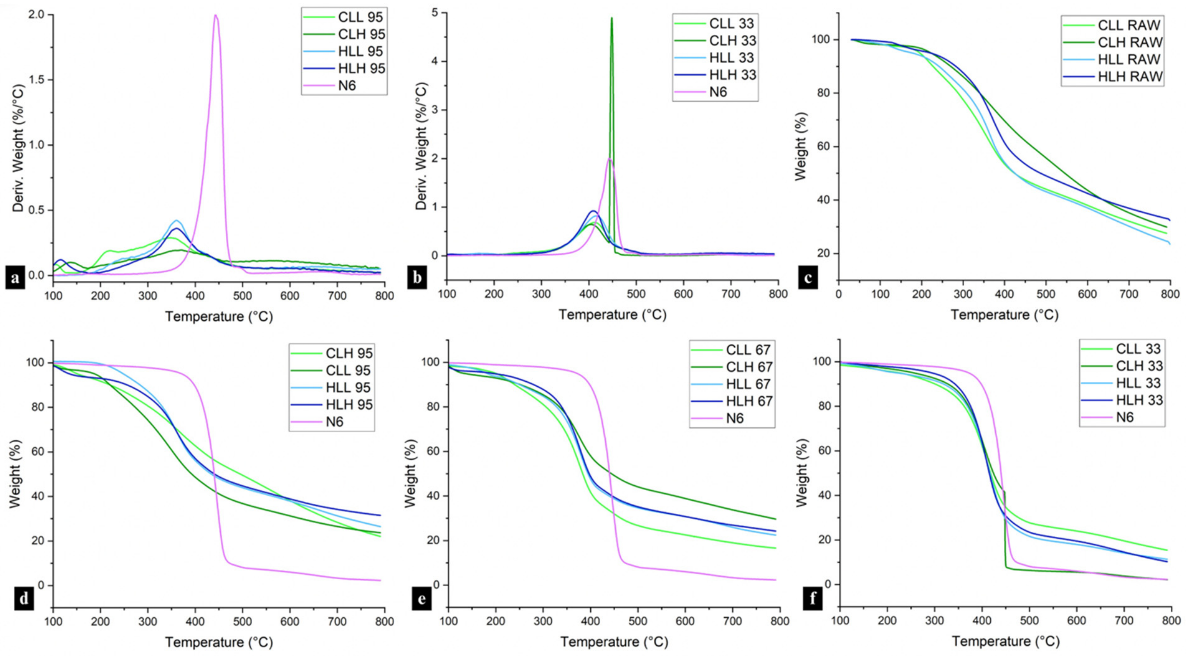

Figure S12. DTG curves of of (a) 95% and (b) 33% lignin/nylon 6 fibers, and decomposition curves of (c) lignin raw material, (d) 95%, (e) 67%, and (c) 33% lignin/nylon 6 fibers.

Table S3: DMA measurements for all samples. Samples with * were too brittle to be mounted and were not measured.

Sample	UTS (MPa)	Elongation (%)	Young's Modulus (MPa)	Avg Stiffness (N/m)	Avg Displacement (um)	Avg Relaxation modulus (MPa)	Creep Compliance (µm²/N)
CLH 95	*	*	*	*	*	*	*
CLH 67	0.43±0.01	23±10	0.17±0.11	2959±1721	171±60	61±45	63968±18275
CLH 33	3.87±0.29	69±44	0.64±0.25	2091±794	696±343	68±13	28999±6977
CLL 95	*	*	*	*	*	*	*
CLL 67	*	*	*	*	*	*	*
CLL 33	5.57±0.68	56±5	1.26±0.27	8240±3093	492±136	118±27	15383±3318
HLL 95	*	*	*	*	*	*	*

HLL 67	*	*	*	*	*	*	*
HLL 33	3.97±1.33	39±14	0.67±0.24	4800±1311	326±110	51±13	26267±4439
HLH 95	*	*	*	*	*	*	*
HLH 67	0.28±0.13	9±7	0.10±0.04	5799±1549	197±85	89±26	179772±130279
HLH 33	2.96±0.12	49±18	0.44±0.20	1876±961	662±219	38±21	49887±31059
N6	4.47±2.11	115±63	0.37±0.15	2209±718	2244±1237	24±6	114838±50488

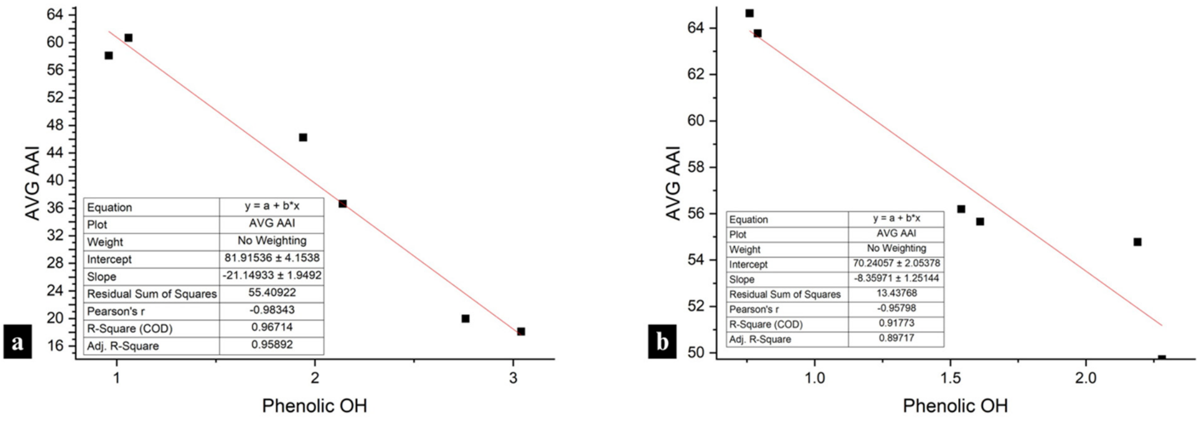

Figure S13: Linear regression plots of initial AAI and phenolic OH content in samples with (a) low M_w lignin, and (b) high M_w lignin.

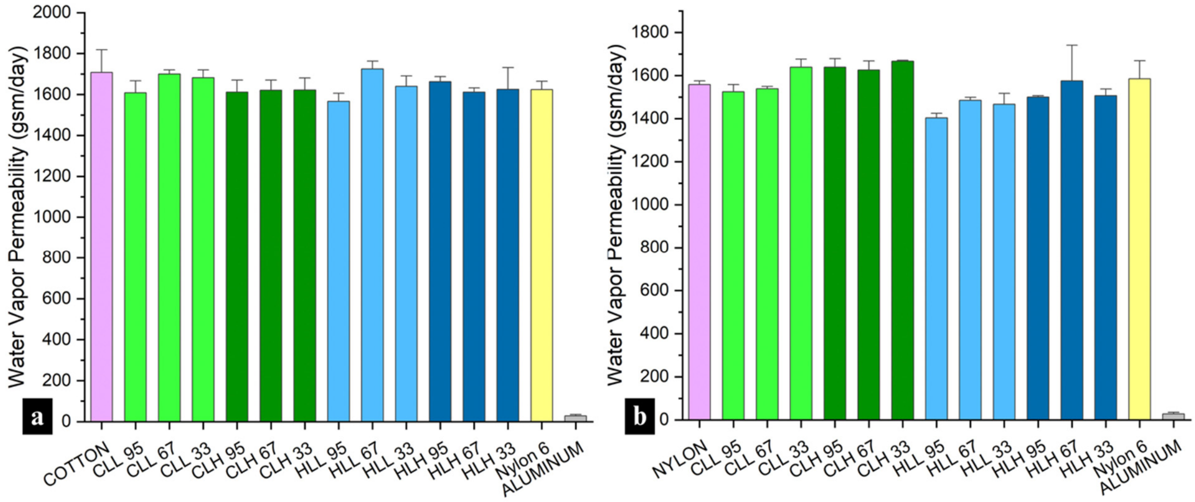

Figure S14. Water vapor permeability of neat substrate, aluminum foil as control, and lignin/nylon 6 and neat nylon 6 nanofiber membranes on (a) cotton fabric substrate, and (b) nylon fabric substrate.

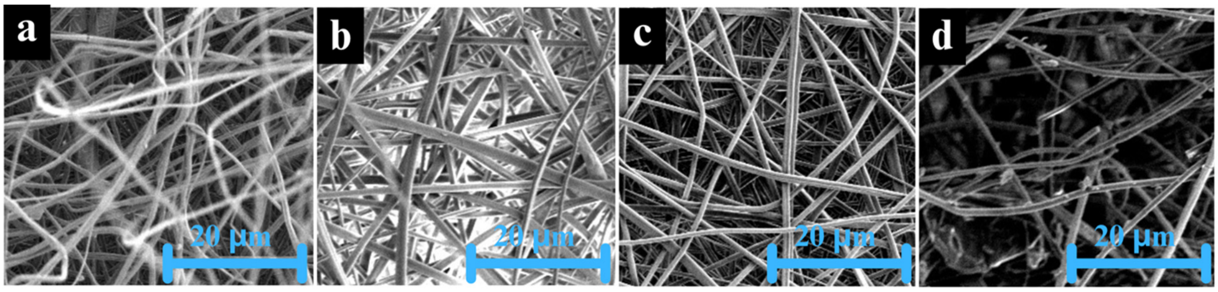

Figure S15: SEM micrographs of nanofiber samples collected from the nylon substrate: a). CLL 2:1, b). CLH 2:1, c). HLL 2:1, d). HLH 2:1.

Table S4: Average water contact angle measurements for all stand-alone nanofiber membranes.

**Sample**	**CLL 95**	**CLL 67**	**CLL 33**	**CLH 95**	**CLH 67**	**CLH 33**	**HLL 95**	**HLL 67**	**HLL 133**	**HLH 95**	**HLH 67**	**HLH 33**	**Nylon 6**
After 10 sec	114 ± 1	110 ± 12	100 ± 6	96 ± 1	94 ± 8	117 ± 4	95 ± 4	86 ± 3	45 ± 16	125 ± 2	150 ± 31	120 ± 6	72 ± 7
After 5 min	112 ± 4	102 ± 6	78 ± 16	56 ± 38	0 ± 0	113 ± 4	112 ± 14	0 ± 0	0 ± 0	112 ± 14	0 ± 0	70 ± 15	0 ± 0
